# Comparative analysis of clinically approved lipid nanoparticles for intranasal siRNA delivery against SARS-CoV-2

**DOI:** 10.1007/s13346-026-02076-y

**Published:** 2026-02-18

**Authors:** Yuan Zhang, Matt D. Johansen, Scott Ledger, Rebecca Shipley, Guanshu Zhao, Ernest Moles, Stuart Turville, Pall Thordarson, Philip M. Hansbro, Maria Kavallaris, Anthony D. Kelleher, Chantelle L. Ahlenstiel

**Affiliations:** 1 Kirby Institute, UNSW, Sydney, NSW 2052 Australia; 2 UNSW RNA Institute, UNSW Sydney, Sydney, 2052 Australia; 3 Centre for Inflammation, Faculty of Science, School of Life Sciences, Centenary Institute and University of Technology Sydney, Sydney, NSW 2050 Australia; 4 Children’s Cancer Institute, Lowy Cancer Research Centre, UNSW Sydney, Sydney, 2052 Australia; 5 Australian Centre for Nanomedicine, Faculty of Engineering, UNSW Sydney, Sydney, 2052 Australia; 6 School of Clinical Medicine, Medicine and Health, UNSW Sydney, Sydney, 2052 Australia

**Keywords:** SARS-CoV-2, SiRNA, Lipid nanoparticles, RNA interference, Drug delivery

## Abstract

SARS-CoV-2 continues to circulate globally, with persistent hospitalizations, despite a successful global vaccination strategy. We have developed highly conserved, antiviral short interfering (si)RNA and demonstrated in vivo antiviral efficacy following intranasal treatment of mice with naked siRNA. To enhance antiviral efficacy and siRNA protection, in this study we investigated the use of LNP packaging to improve delivery and efficacy. We examined three clinically approved lipid nanoparticle (LNP) formulations that mimic the compositions of Alnylam’s Onpattro (MC3), Moderna’s Spikevax (SM-102), and Pfizer-BioNTech’s Comirnaty (ALC-0315) RNA-based therapeutics, to identify the optimal formulation for antiviral siRNA therapeutic respiratory delivery and antiviral efficacy. All LNP formulations assessed showed successful delivery of siRNA to respiratory cells in vitro and provided effective silencing of siRNA targeted SARS-CoV-2 genes. However, the MC3-based LNP-siRNA (MC3 LNP-siRNA) treatment elicited the least off-target immune activation, with no induction of interferon stimulated genes. Additionally, the MC3 LNP-siRNA remained effective when administered 24 h post-infection, significantly reducing viral RNA levels in vitro. Chemical modification of siRNA with 2′‑O‑methyl incorporation further attenuated immune activation, without compromising efficacy. In vivo intranasal delivery of MC3 LNP-siRNA was generally well tolerated, with no adverse effects on body weight or pulmonary function at therapeutic doses, although mild pulmonary leukocyte infiltration was observed at higher or repeated doses. Our study demonstrates that LNP-encapsulated and chemically modified siRNAs can provide an effective and mutation-resilient antiviral strategy. This study compares clinically relevant LNP formulations for siRNA delivery to the respiratory tract, demonstrating that MC3-based LNPs offer a promising platform for safe and effective RNA therapeutic delivery.

**Supplementary Information:**

The online version contains supplementary material available at https://doi.org/10.1007/s13346-026-02076-y.

## Introduction

Severe Acute Respiratory Syndrome-Coronavirus 2 (SARS-CoV-2), the pathogen causing COVID-19, has been found in the respiratory tract, heart, kidney, and other tissues and organs of COVID-19 patients [1]. Prevention of severe COVID-19 disease relies on host immunity following vaccination or previous infection. COVID-19 vaccines provide strong protection against severe outcomes [2, 3], but this immune protection diminishes within three to six months following booster doses, especially in older or immunocompromised individuals [4]. Circulating viral mutations further contribute to ongoing infections.

Several therapies have been developed for COVID-19, including antivirals (e.g. Paxlovid, Lagevrio, Veklury) and monoclonal antibodies [5–9]. However, each has limitations. Paxlovid (nirmatrelvir/ritonavir) interacts with other drugs and reduces protein levels, making it unsuitable for patients with cirrhotic hypoproteinemia or malnutrition [10]. Veklury (Remdesivir) can elevate liver enzymes [11, 12], while Lagevrio (Molnupiravir) shows lower efficacy, higher mortality, and adverse effects on bone and cartilage [13, 14]. Monoclonal antibodies are often compromised by viral mutations, and effective delivery to target organs remains challenging [15]. These limitations highlight the need for safe, broad-spectrum antiviral platforms.

Small interfering RNAs (siRNAs) represent a promising therapeutic strategy due to their high specificity and potent antiviral activity. In our previous work, we screened 39 siRNA candidates targeting conserved regions of the SARS-CoV-2 genome (UTR, ORF1ab, Spike, Envelope, Membrane and Nucleocapsid) and identified four highly conserved siRNAs targeting distinct regions of the SARS-CoV-2 genome with broad-spectrum antiviral efficacy against multiple SARS-CoV-2 variants of concern (VOCs) [16]. Moreover, incorporating specific chemical modifications (e.g., 2’-O-methyl) significantly reduced immunostimulatory off-target effects by minimizing interferon-stimulated gene (ISG) activation [17, 18]. However, the efficiency of therapeutic delivery to non-hepatic target sites remains a challenge [19]. Here, we investigate lipid nanoparticle (LNP) encapsulation of four previously identified lead candidate COVID siRNAs, siRNA18, siRNA25, siRNA27, and siRNA30, targeting conserved SARS-CoV-2 regions (Membrane, Nucleocapsid, and ORF1a/NSP1). These siRNAs demonstrated in vitro antiviral activity with nanomolar potency, establishing them as lead candidates for evaluation with clinically approved LNP formulations [20]. Delivery of siRNA was assessed using three lipid-based nanoparticle (LNP) formulations differing in their ionisable lipid component, including D-Lin-MC3-DMA (used in Onpattro, the first FDA-approved siRNA therapy), SM-102 (used in Spikevax COVID-19 mRNA vaccine), and ALC-0315 (used in Comirnaty COVID-19 mRNA vaccine); resulting siRNA-loaded LNP formulations are herein abbreviated as MC3, SM-102 and ALC-0315, respectively. These formulations were selected based on their established clinical safety profiles and proven efficacy in nucleic acid delivery, making them promising candidates for siRNA encapsulation [2, 21, 22]. Notably, as the only three ionizable cationic lipids currently approved for clinical RNA therapeutics, these ionizable lipids differ in their pKa and structure, which might influence their charge potency and immune reactivity. The principal distinctions among them lie in their headgroup chemistry and linkage patterns: DLin-MC3-DMA contains two tertiary amine groups, whereas ALC-0315 and SM-102 possess alcohol–ester frameworks, with SM‑102 possessing longer side chains and a more complex cyclic moiety. Owing to these molecular differences, DLin-MC3-DMA tends to exhibit a stronger cationic profile that enhances RNA complexation, while the greater hydrophobicity of ALC-0315 and SM-102 may facilitate membrane fusion during cellular uptake [23]. Studies have also shown that, at higher systemic doses (5 mg/kg), ALC-0315-based LNPs elevate hepatic toxicity markers such as ALT and bile acids, whereas MC3-based LNPs do not produce such effects [24]. These distinctions are particularly relevant for respiratory delivery, where minimizing mucosal irritation and off-target inflammation is essential to ensure safety and tolerability.

Gene silencing efficacy of these LNP-encapsulated siRNAs (LNP-siRNAs) was evaluated using SARS-CoV-2 GFP reporter cell lines and their antiviral efficacy was assessed using live SARS-CoV-2 infection. Both gene silencing and off-target immune activation assays were performed in Calu-3 cells—a human bronchial epithelial cell line that closely mimics the physiological environment of the respiratory epithelium and is highly relevant for studying respiratory viruses [25–27]. We further investigated specific chemical modification to identify the potential for decreasing the ISG-related off-target response to LNP formulations. Finally, the in vivo tolerability of MC3 LNP-siRNA was evaluated via intranasal administration directly to the respiratory tract. Overall, this study will help define the optimal LNP composition and siRNA modification strategy for safe and effective respiratory delivery and provides a foundational step toward the clinical translation of directly delivered siRNA-based therapeutics for respiratory viral infections.

## Methods

### Animals

All animal experiments were conducted in accordance with the Australian Code for the Care and Use of Animals for Scientific Purposes (8th edition, 2013; updated 2021, National Health and Medical Research Council, Australia) and were approved by the Sydney Local Health District (SLHD) animal ethics committee under approval AEC 2020-019. Institutional Biosafety Committee (IBC) approval was provided by the SLHD IBC under approval IBC 20–051. C57BL/6J female mice were selected for these studies as a widely used immunocompetent background strain, which is suitable for pulmonary safety and tolerability assessments in this study. A total of 30 C57BL/6J female mice (8 weeks of age) were purchased from Australian Bioresources (Mossvale, NSW). Mice were housed under standard 12hr light:dark cycles with *ad libitum* access to standard chow and water. Mice were transported to Centenary Institute and acclimatised for 1 week prior to experimentation.

### Cell culture

HEK293T cells (ATCC^®^, CRL-3216, Rockville, MD, USA) were cultured in Dulbecco’s modified Eagle’s medium (DMEM, Gibco^TM^ Cat#11995065) with 10% heat-inactivated fetal bovine serum (FBS) and 100 U/mL Penicillin-Streptomycin (Pen-Strep, Gibco^TM^ Cat#15140122). HeLa T4+ cells (NIH AIDS Reagent Program, ARP-154, USA) were cultured in DMEM with 10% heat-inactivated FBS, 100 U/mL Pen-Strep and 500 µg/mL G418 (Gibco^TM^ Cat#10131027). Vero E6 cells (ATCC^®^, CRL-1586, Rockville, MD, USA) were cultured in Eagle’s Minimum Essential Medium (EMEM) with 10% heat-inactivated FBS, 1 mM sodium pyruvate (Gibco^TM^ Cat#11360070), and 100 U/mL Pen-Strep. Calu-3 cells (ATCC^®^, HTB-55, Rockville, MD, USA) were cultured in Dulbecco’s Modified Eagle Medium/Nutrient Mixture F-12 (DMEM/F-12, Gibco^TM^ Cat#11039021) with 10% heat-inactivated FBS, 1% non-essential Amino Acid (AA, Sigma Cat# M7145), and 1% Glutamax (Gibco^TM^ Cat#35050079). All cells were cultured at 37^*◦*^C with 5% CO_2_.

### Viruses

The virus isolates used in both in vitro and in vivo siRNA screening include those below and were kindly supplied by A/Prof Stuart Turville.

hCoV-19/Australia/NSW4605/2021 (EPI_ISL_1911187); Delta

hCoV-19/Australia/NSW-RPAH-1933/2021 (EPI_ISL_6814922); BA.1

hCoV-19/Australia/NSW-ICPMR-44477/2023 (EPI_ISL_17408981); XBB.1.5

### Formulation and synthesis of LNP-siRNAs

All siRNA and chemically modified siRNAs were synthesized by the UNSW RNA Institute. LNP-siRNAs were formulated with 60 mM ionizable lipid (D-Lin-MC3-DMA, Assay Matrix, Cat# HY-112251; SM-102, Assay Matrix, Cat# HY-134541; or ALC-0315, Assay Matrix, Cat# HY-138170), 20 mM 18:0 PC (DSPC; Sigma-Aldrich, Cat#850365P), 20 mM Cholesterol (Sigma-Aldrich, Cat# C8667), 5 mM DMG-PEG-2000 (Sigma-Aldrich, Cat#880151P), and 2 mM 18:1 Liss Rhod PE (Sigma-Aldrich, Cat# 810150P). Formulations are shown in Table 1. LNP-siRNAs were formed as previously described [28]. Briefly, siRNA was dissolved in 25 mM sodium acetate, pH 4, with the nitrogen to phosphate ratio of 3.2. Organic (lipid) and aqueous (siRNA) phases were combined using a NanoAssemblr Ignite microfluidic mixer (Precision NanoSystems Inc.) at a 1:3 (vol:vol) ratio and a total flow rate of 12 mL/min. For long-term storage, resulting LNP-siRNAs were buffer exchanged into Tris-Sucrose. Briefly, LNP-siRNAs were diluted 1:40 into Tris-Sucrose buffer (20 mM Tris and 8.7% w/v sucrose with pH7.4) and subsequently concentrated using 15 mL Amicon 10 K centrifugal filter units (2000 g for 10 min at 20^*◦*^C, Merck, UFC901008). LNPs-siRNAs were filter-sterilized (0.22 *µ*M) and stored at −30^*◦*^C.

**Table 1:** Molar ratio composition of the three LNP-siRNA formulations

Components*	Molar ratios [29]		
	Anylam’s Onpattro [21]	Moderna’s Spikevax [2]	Pfizer-BioNTech’s Comirnaty [22]
Ionizable lipid	50% (D-Lin-MC3-DMA)	50% (SM-102)	50% (ALC-0315)
DSPC (18:0 PC)	10%	10%	10%
Cholesterol	38%	38%	38%
DMG-PEG-2000	1.5%	1.5%	1.5%
18:1 Liss Rhod PE	0.5%	0.5%	0.5%

*All components were dissolved initially in pure ethanol

### LNP-siRNA encapsulation efficiency and nano-size measurement

Encapsulation efficiency (EE%) and encapsulated siRNA concentrations were measured using the Quant-iT^TM^ RiboGreen RNA Assay Kit according to the manufacturer’s protocol. Briefly, LNP-siRNAs were diluted in TE Buffer and added to the 96-well plate with or without Triton X-100. Plates were read using a CLARIOstar Plus (BMG Labtech) with the excitation and emission wavelength of 485 nm and 528 nm after adding Ribogreen (1:100) and incubating in 30℃ for 15 min. EE% and encapsulated siRNA concentrations were then calculated according to the linear standard curves.

For nano-size measurement, samples were diluted in UltraPure DNase/RNase-Free water in Square Polystyrene Cuvettes (ATA Scientific, Cat# DTS0012) before measuring zeta potential using the Zetasizer (Malvern Panalytical), after which Nano-sizes and polydispersity index (PI%) were recorded.

### Construction and verification of Calu-3 SARS-CoV-2 lentiviral-GFP-reporter cell lines

Synthetic SARS-CoV-2 DNA fragments of ORF1a-Nsp1 (nt 266–805), Membrane (nt 26,523–27,191), and Nucleocapsid (nt 29,200–30,460), were synthesised (Genewiz from Azenta Life Sciences) based on the reference genome *SARS-CoV-2 Wuhan-Hu-1* (GenBank accession NC_045512.2),and were cloned into the FG11F lentiviral constructs expressing individual fragments driving GFP for the 5`UTR or fused to GFP for the remaining fragments were generated via conventional cloning strategies.

Lentivirus was generated using a second-generation lentiviral system, as previously described [30–32]. In brief, the lentiviral vector DNA, psPAX2 packaging, and VSV-G envelope plasmid DNA were combined at a ratio of 4:4:1. After 72 hours of culturing, Virus-containing media (VCM) was harvested, and viral stock titres were then analysed. Calu-3 cells were then infected with Lentiviral-SARS-CoV-2-GFP, then Blasticidin selection (20µg/mL) and cell sorting were performed to acquire Lenti-SARS-CoV-2- GFP-reporter cell lines with the relatively higher expression rates of GFP. All lentiviral constructs were verified by Sanger sequencing prior to virus production to ensure sequence fidelity.

To verify Lenti-SARS-CoV-2-GFP reporter cell lines, candidate siRNA- 18, 25, and 30 with the concentration of 5 nM were transfected into their respective Calu-3 reporter cell lines using Lipofectamine RNAi MAX (Invitrogen^TM^ Cat#13778100). GFP expression was then analysed using flow cytometry to identify the downregulation effect of candidate siRNAs on their targeted genes of reporter cells.

### Transfection of siRNA and modified siRNA using lipofectamine

All Kirby COVID siRNAs used in this study were synthesised at the UNSW RNA Institute. Transfection of siRNAs (5 nM or as indicated) was performed using Lipofectamine RNAiMAX (Invitrogen™, Cat#13778100) according to the manufacturer’s protocol. Cell fixation and flow cytometry were conducted on designated days. To evaluate potential improvements in immunological safety, selected siRNAs were chemically modified with 2'-O-methylation at positions 4, 5, and 6 of the antisense strand (Ome-AS456). This modification was designed based on its reported ability to reduce off-target immune activation while preserving gene-silencing efficacy [17, 18, 33–35]. The sequences of all siRNAs and their respective modifications are summarised in Table 2**.**

**Table 2:** Sequences of Kirby COVID siRNAs

Modified siRNAs	Strand	Sequence (5'—3')	Target genes
siRNA Scram [16]	Sense Antisense	CGCUGGGACGUGUGCCUGA [dT][dT] UCAGGCACACGUCCCAGCG [dT][dT]	-
siRNA 18 [16]	Sense Antisense	GCUACAUCACGAACGCUUU [dT][dT] AAAGCGUUCGUGAUGUAGC [dT][dT]	Membrane
siRNA 25 [16]	Sense Antisense	GGGUUGCAACUGAGGGAGC [dT][dT] GCUCCCUCAGUUGCAACCC [dT][dT]	Nucleocapsid
siRNA 27 [16]	Sense Antisense	CGAGAAAACACACGUCCAA [dT][dT] UUGGACGUGUGUUUUCUCG [dT][dT]	ORF1ab (NSP1)
siRNA 30 [16]	Sense Antisense	GGCAUUCAGUACGGUCGUA [dT][dT] UACGACCGUACUGAAUGCC [dT][dT]	ORF1ab (NSP1)
siRNA 30 mod* [18, 33–35]	Sense Antisense	GGCAUUCAGUACGGUCGUA [dT][dT] UACmGmAmCCGUACUGAAUGCC [dT][dT]	ORF1ab (NSP1)

*mod: Ome-AS4,5,6 (2'O-Methylation at positions 4, 5, and 6)

### Flow cytometry and cell sorting

After resuspending the cells and centrifuging (300g, 5 min), cells were fixed in 2% of paraformaldehyde for 30 min at room temperature. Flow cytometry was then performed using the BD LSRFortessa SORP with at least 20,000 cells per sample. For cell sorting, cells were resuspended and centrifuged (300g, 5 min), then resuspended again in Flow Cell Sorting buffer (PBS, EDTA 2mM, heat-inactivated FBS 10%) before filtration using 5mL falcon tubes with cell strainer snap cap. After cell sorting, sorted cells were collected using 2-5mL cell media with 20% heat-inactivated FBS.

### Off-target effect of siRNAs and LNP-siRNAs

To assess potential off-target immune responses, we quantified the expression of interferon–stimulated genes (ISGs) in HeLa T4⁺ and Calu-3 cells following treatment with LNP-formulated siRNAs. HeLa T4⁺ cells were used to assess four canonical ISGs—ISG20, Viperin, IFIT1, and OAS1 [36]—while Calu-3 cells were additionally assessed for RIG-I. HeLa T4⁺ and Calu-3 cells were seeded at 4 × 10^4^ cells per well into 48-well plates and transfected with 5 nM naked siRNAs or 50 nM LNP-formulated siRNAs. Transfections were performed 6 hours post-seeding for HeLa T4⁺ cells and 48 hours post-seeding for Calu-3 cells. Positive controls included IFN-β (500 IU/mL) added 24 hours prior to RNA harvest, and Poly(I:C) (0.1 μg/mL), transfected 6 hours before collection using Lipofectamine RNAiMAX, per manufacturer’s instructions. Cells were lysed 72 hours post-treatment for RNA extraction. Total RNA was extracted using the Monarch Total RNA Miniprep Kit (NEB, Cat#T2010S), and relative mRNA expression levels were determined by SYBR Green–based RT-qPCR (Luna® Universal One-Step RT-qPCR Kit, E3005), using GAPDH as the housekeeping gene. Primers were designed to specifically amplify the target transcripts as follows: IFIT1 (forward 5′-AACTTAATGCAGGAAGAACATGACAA-3′, reverse 5′-CTGCCAGTCTGCCCATGTG-3′), OAS1 (forward 5′-AGGTGGTAAAGGGTGGCTCC-3′, reverse 5′-ACAACCAGGTCAGCGTCAGAT-3′), ISG20 (forward 5′-TCGTTGCAGCCTCGTGAAC-3′, reverse 5′-TCCCTCAGGCCGGATGA-3′), Viperin (forward 5′-GTGAGCAATGGAAGCCTGATC-3′, reverse 5′-GCTGTCACAGGAGATAGCGAGAA-3′), RIG-I (forward 5′-AAAGCCAGTGGAGATCACAATA-3′, reverse 5′-TGATGACATTGCCCACATACT-3′), and GAPDH (forward 5′-CACTGCCAACGTGTCAGTG-3′, reverse 5′-CAGCGTCAAAGGTGGAGGA-3′).

### Protective effect of siRNAs and LNP-siRNAs against SARS-CoV-2

VeroE6 and Calu-3 cells were seeded into 96-well-plate at 1.5×10^4^/well and 4×10^4^/well, respectively, and transfected with siRNAs at 5 nM (Lipofectamine) or 25–50 nM (LNP formulations), administered either 2 h pre-infection, 2 h post-infection, or 24 h post-infection, as indicated. Supernatants were collected after cell survival assay on Day 5 to Day 7 post-infection. RNA extraction (Monarch Total RNA Miniprep kit, Cat# T2010S) and qRT-PCR (Luna® Uni- versal Probe One-Step RT-qPCR Kit, E3006) were then performed to test the viral loads according to the manufacturer’s protocol.

### Cell survival assay

Cells were stained using NucBlue (Invitrogen^TM^ Cat#R37605) according to manufacturer’s protocol 24 hours prior to imaging, then images were acquired for brightfield (white light) and blue light spectrums by IN Cell Analyzer 2500 HS plate reader with 10× zoom lens. Nuclei counts were quantified using IN Carta Image Analysis Software to estimate viable cell numbers, as detached or non-nucleated cells were excluded during imaging. This method thus provided a relative measure of cell viability rather than a metabolic survival assay. All experiments were conducted in triplicate or six replicates where indicated.

### In vivo biosafety assessment of intranasally delivered LNP-siRNA

For single-dose evaluations, C57BL/6J mice were divided into four groups (n = 6 per group), anaesthetised with isoflurane and treated intranasally on Day 0 with either 1 µg, 2.5 µg, or 5 µg siGFP encapsulated in MC3 LNP-siRNA formulations or PBS vehicle as a negative control (PBS). Intranasal dosing was performed under light isoflurane anaesthesia with mice placed in a supine position. A total volume of 30 µL (15 µL per nostril) of the LNP-siRNAs was administered dropwise using a calibrated micropipette. The experiment endpoint was Day 5, and bronchoalveolar lavage fluid (BALF) was collected for leukocyte and differential cell count enumeration. For repeated-dose studies, mice were divided into two groups (n = 8 per group) and administered 5 µg of MC3 LNP-siGFP intranasally, once daily for five consecutive days (Day 0 to Day 4), with PBS-treated mice as controls. Body weights were recorded daily. At the day 5 endpoint, mice were surgically anaesthetised and tracheotomised for lung function analysis using the FlexiVent apparatus (SciReq, Canada). Pulmonary function parameters, including inspiratory capacity (IC), resistance of the respiratory system (Rrs), resistance of central airways (Rn), hysteresis area, forced vital capacity (FVC), and total lung capacity (TLC), were measured as previously described [37]. BALF was also harvested to measure leukocyte and differential cell counts in the lungs.

### Statistical analysis

Data are presented as mean ± standard deviation (SD) from at least three independent biological replicates. Statistical analyses were performed with GraphPad Prism software (version 10.2.3). For comparisons between two independent groups, an unpaired two-tailed Student’s t-test was applied. For comparisons involving more than two groups, one-way analysis of variance (ANOVA) was applied when data met the assumptions of approximate normality and homogeneity of variance. Given the analyses based on three biological replicates, where formal normality testing may be underpowered,non-parametric alternatives would be considered if distributional assumptions were not met. Normality was assessed using a combination of Shapiro–Wilk testing and visual inspection of Q–Q plots. Specifically, Tukey’s multiple comparisons test was used following one-way ANOVA for all pairwise group comparisons, while Dunnett’s multiple comparisons test was applied when comparing multiple treatments to a single control. For experiments involving two independent variables, two-way ANOVA was performed, followed by Šídák’s multiple comparisons test. For mouse experiments, body weight data were analysed using two-way ANOVA, while leukocyte counts and differential cell counts were analysed using one-way ANOVA. * p < 0.05, ** p < 0.01, *** p < 0.001, **** p < 0.0001.

## Results

### Preparation of LNP-siRNAs and exploration of cell entry properties

**Fig. 1:**
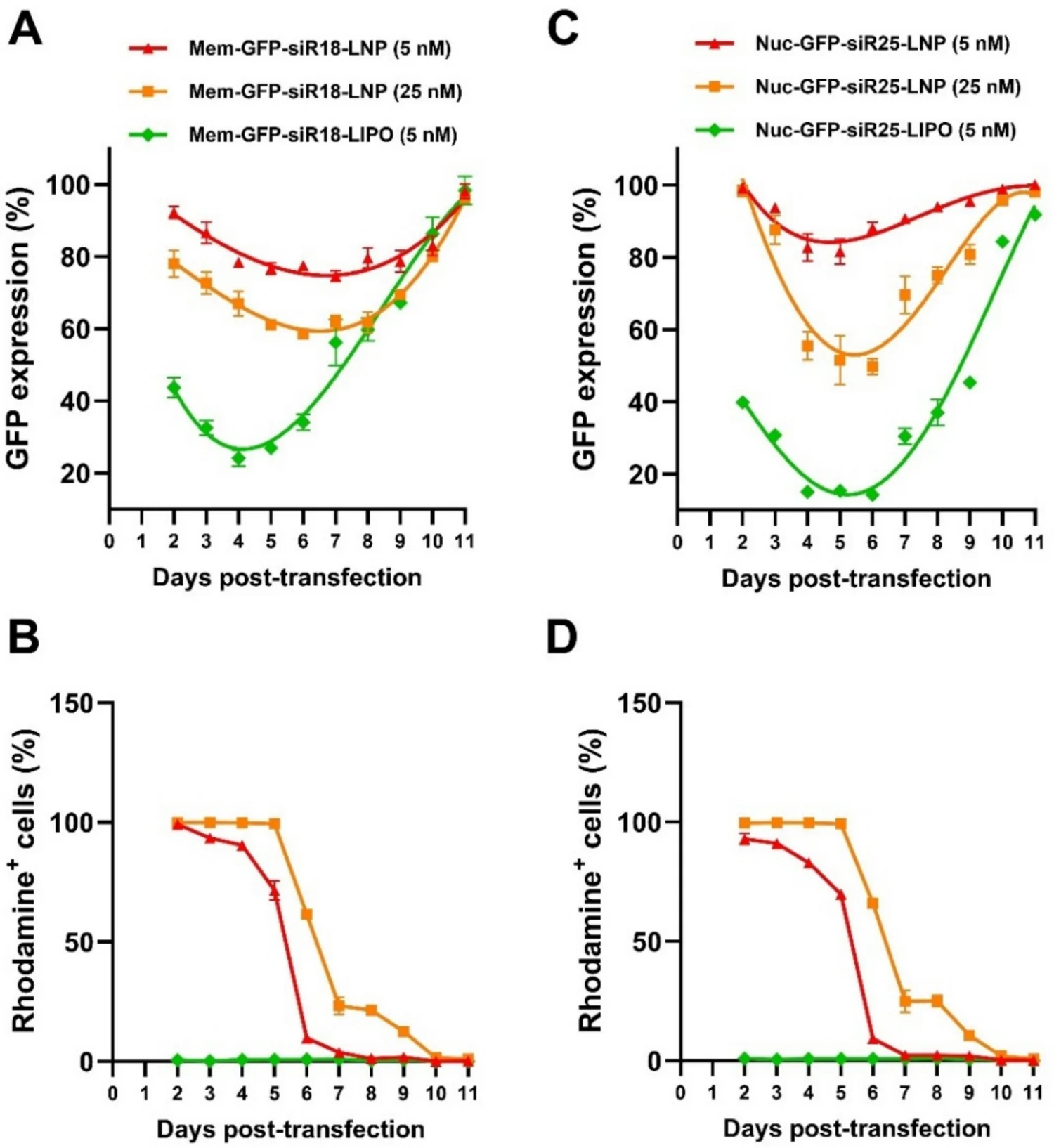
Down-regulation of GFP expression in 293 T reporter cell-lines expressing GFP fused SARS-CoV-2 proteins after transfection with MC3-LNP-siRNAs. Cells were seeded at 3 × 10^4^ cells per well in 48-well plates, and LNP-siRNAs (18 and 25) were added the following day. Cells were fixed and analysed by flow cytometry to assess GFP expression, using the B-530 channel to detect GFP fluorescence and the YG-585 channel to identify the percentage of rhodamine-positive (PE) cells. GFP expression was quantified by normalizing the percentage of GFP-positive cells in each sample to that of untreated reporter cells. The PE signal reflects the presence of labelled lipid only and does not represent the persistence of the siRNA cargo. Mem-GFP expression levels (**A**) and percentage of LNP-positive cells (**B**) after transfection with 5 nM or 25 nM LNP-siRNA 18, or Lipofectamine-siRNA 18 in 293 T Mem-GFP reporter cell line. (**C**)(**D**) Nuc-GFP expression levels (C) and percentage of LNP-positive cells (D) in 293 T Nuc-GFP reporter cells after transfection with 5 nM or 25 nM LNP-siRNA 25, or Lipofectamine-siRNA 25 in the 293 T Nuc-GFP reporter cell line. All experiments were performed in triplicate. Data were presented as mean ± standard deviation (SD).

We investigated whether four lead anti-COVID siRNAs could be successfully delivered into cells when encapsulated in a lipid nanoparticle (LNP). As the first FDA-approved siRNA delivery system for therapeutics, rhodamine labelled LNPs reproducing D-Lin-MC3-DMA (MC3) LNP formula were used as proof-of-concept. 293 T Mem-GFP and Nuc-GFP reporter cell lines were used to assess siRNA-mediated gene silencing **(**Fig.S1**)**. In these cells, GFP is not the target of silencing but serves as a reporter to indicate the effectiveness of siRNAs targeting membrane (Mem) or nucleocapsid (Nuc) regions, respectively. We observed that a 5 nM LNP-siRNA treatment was not sufficient to significantly suppress gene expression and required a higher dose of 25 nM to achieve GFP suppression similar to the lipofectamine control transfection of 5 nM siRNA **(**Fig. 1**)**. Maximal suppression of gene expression was reported in Lipofectamine-siRNA-treated cells on day 4, 58.89% for Mem-siRNA 18 and 43.73% for Nuc-siRNA 25, respectively, but GFP expression recovered rapidly thereafter, consistent with the transient nature of Lipofectamine-mediated transfection and shorter intracellular stability of unencapsulated siRNAs. In comparison, cells treated with 25 nM LNP-siRNA showed the greatest decrease in GFP expression on day 6: 62.13% for Mem-siRNA 18 and 55.53% for Nuc-siRNA 25, respectively **(**Fig. 1A and 1C). Following transfection, over 90% of cells were Rhodamine-labeled LNP-positive on both Day 2 and Day 3 for both 5 nM and 25 nM LNP-siRNAs. This high percentage, as indicated by PE fluorescence, remained above 70% through Day 5 (5 nM) and Day 6 (25 nM), reflecting a sustained association of LNPs with cells over multiple days. However, by Day 7, a sharp decline in LNP-positive cells was observed in both 5 nM and 25 nM LNP-siRNA, potentially due to a combination of factors including exhaustion of extracellular LNPs, degradation of LNPs over time, and the fluorescence decay of rhodamine (Fig. 1B and 1D).

### Characterisation and gene-silencing effect of LNP-siRNA formulations

To identify an optimal LNP formulation for delivery of our siRNAs, we tested additional formulations based on LNPs used in FDA-approved COVID-19 mRNA vaccines, including Spikevax’s SM-102-based and Comirnaty’s ALC-0315-based formulas, and compared their gene silencing performance to MC3 LNP formula. Characterisation and quality control of batches included quantifying the nano-size, PI%, percentage of encapsulation efficiency (EE%) and the encapsulated siRNA concentration (Table 3). Size of MC3 LNP-siRNAs was 78.9–86.3.9.3 nm with PDI% of ≤0.23, while SM-102 LNP-siRNAs were 83.2–93.6.2.6 nm with PDI% of ≤0.18 and ALC-0315 LNP-siRNAs were 97.1–109.3 nm with PDI% of ≤ 0.16. These values are consistent with the recommended range (≤150 nm, PDI% ≤ 0.3) for pulmonary delivery [38, 39]. The percentage of EE% of MC3 LNP-siRNAs, SM-102 LNP-siRNAs and ALC-0315 LNP-siRNAs were all higher than 80%, which aligns with the thresholds for selecting nano formulations for further study (Table. 3). Specifically, the mean total and encapsulated siRNA concentrations for MC3 LNP-siRNAs were 112.9 ± 12.5 µg/mL and 104.5 ± 10.9 µg/mL, respectively, yielding an average EE% of 92.5 ± 0.8%. For SM-102 LNP-siRNAs, the total and encapsulated siRNA concentrations were 113.4 ± 16.5 µg/mL and 95.2 ± 16.4 µg/mL, corresponding to an EE% of 83.7 ± 2.5%. Similarly, ALC-0315 LNP-siRNAs showed mean total and encapsulated siRNA concentrations of 112.2 ± 10.1 µg/mL and 94.9 ± 10.1 µg/mL, with an EE% of 84.4 ± 1.5% (Table. 3). Representative size distribution plots of all LNP formulations listed in Table 3 are provided in Figure S2, demonstrating narrow, single intensity peaks consistent with homogeneous particle populations.

**Table 3:** Encapsulation efficiency, concentrations, particle size, and polydispersity of MC3, SM-102, and ALC-0315-based LNP-siRNA formulations

LNP-siRNAs ^#^	Total siRNA (ug/mL)	Encapsulated siRNA (ug/mL)	Encapsulation Efficiency (%) (≥ 80%) *	Nano-size (nm) (≤ 150) *	Polydispersity Index (PDI%) (≤ 0.3) *
MC3 LNP Sc	106.7	99.4	93.1	86.1 ± 2.0	0.22 ± 0.01
MC3 LNP 18	131.8	120.6	91.5	86.3 ± 1.1	0.23 ± 0.02
MC3 LNP 25	119.4	110.5	92.6	82.7 ± 2.2	0.23 ± 0.02
MC3 LNP 27	102.1	94.0	92.0	78.9 ± 1.6	0.20 ± 0.04
MC3 LNP 30	104.7	97.8	93.4	81.6 ± 3.7	0.23 ± 0.02
SM-102 LNP Sc	128.6	110.3	85.8	87.9 ± 1.5	0.10 ± 0.02
SM-102 LNP 18	124.0	105.9	85.4	83.2 ± 0.4	0.13 ± 0.01
SM-102 LNP 25	121.8	103.7	85.1	87.2 ± 1.4	0.16 ± 0.04
SM-102 LNP 27	103.2	84.5	81.9	83.8 ± 0.9	0.17 ± 0.03
SM-102 LNP 30	89.6	71.8	80.1	93.6 ± 6.6	0.17 ± 0.04
ALC-0315 LNP Sc	99.3	82.4	83.0	106.2 ± 1.6	0.12 ± 0.02
ALC-0315 LNP 18	126.4	109.0	86.2	97.1 ± 2.3	0.11 ± 0.03
ALC-0315 LNP 25	115.1	97.3	84.5	109.3 ± 2.8	0.16 ± 0.02
ALC-0315 LNP 27	113.7	97.3	85.6	97.9 ± 2.8	0.14 ± 0.01
ALC-0315 LNP 30	106.7	88.4	82.8	100.7 ± 2.5	0.13 ± 0.02

# The numbers following the LNP formulations (18, 25, 27, and 30) denote distinct antiviral siRNA sequences (siRNA 18, siRNA 25, siRNA 27, and siRNA 30) used in this study; Sc, scrambled siRNA control.

* Expected range: Encapsulation efficiency ≥ 80%, size ≤ 150 nm, PDI ≤ 0.3.

**Fig. 2:**
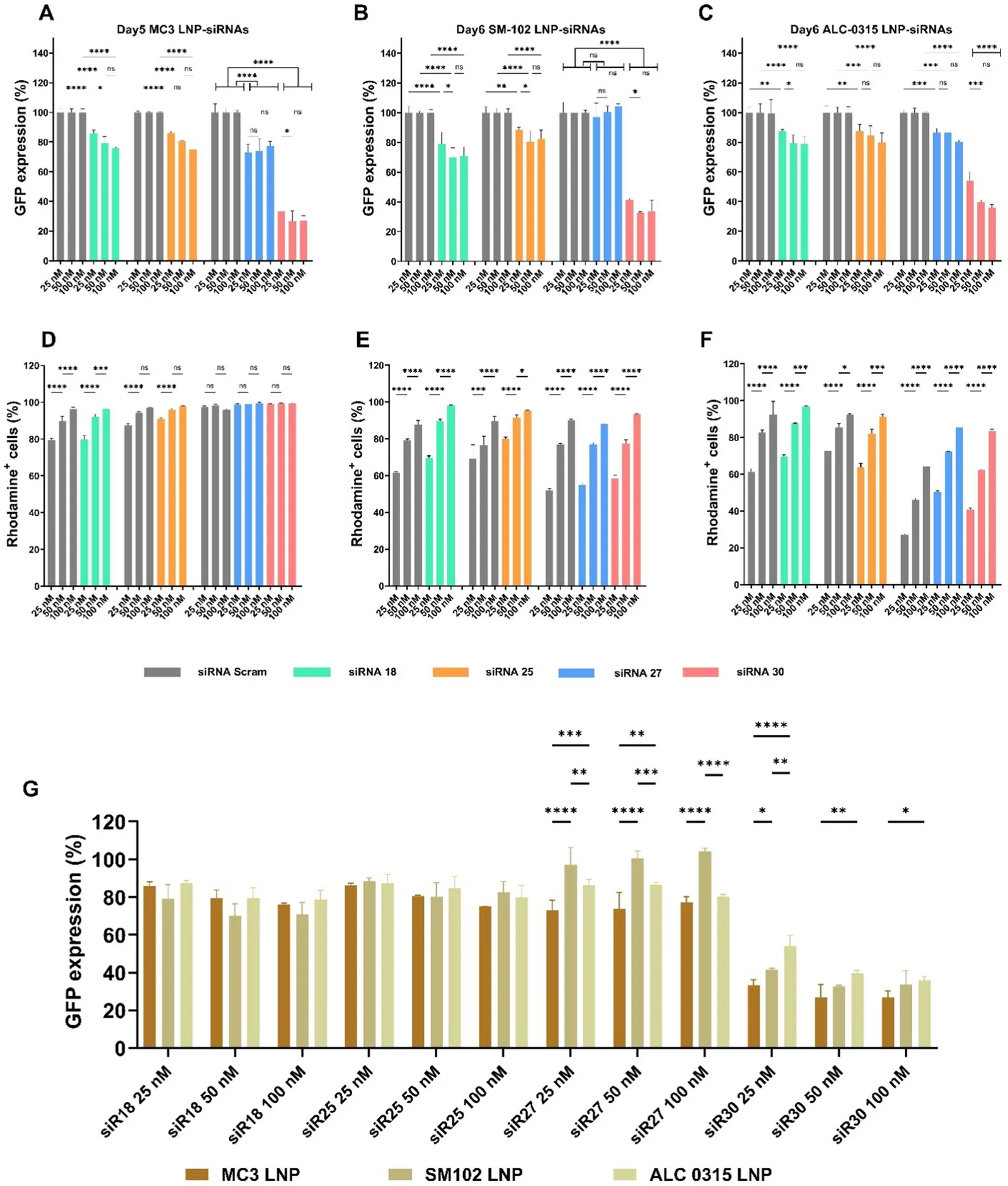
Gene silencing effect of LNP-siRNA in Calu-3 GFP reporter cell lines. Calu-3 Mem, Nuc and Nsp1 GFP reporter cell lines were seeded at 4 ×10^4^/well in 48-well plates and LNP-siRNAs 18, 25, 27 and 30 were added 48 h after cell seeding. Cells were fixed and flow cytometry was performed post transfection with the channel of B-530 for GFP expression and YG-585 for LNP positive cells. **(A–C)** Maximal gene silencing observed at day 5 post-treatment with MC3 LNP-18, MC3 LNP-25, MC3 LNP-27, and MC3 LNP-30 **(A)**, day 6 post-treatment with SM-102 LNP-18, SM-102 LNP-25, SM-102 LNP-27, and SM-102 LNP-30 **(B)** and ALC-0315 LNP-18, ALC-0315 LNP-25, ALC-0315 LNP-27, and ALC-0315 LNP-30 **(C)** in their matching Calu-3 GFP reporter cell line. **(D–F)** Percentage of LNP-positive cells in corresponding Calu-3 reporter cells on Day 5 or 6 post-treatment using MC3 **(D)**, SM-102 **(E)**, or ALC-0315 **(F)** LNP-siRNA formulations. All the results were normalized with LNP-siRNA-Scramble with the corresponding concentrations. Each reporter line expresses a unique SARS-CoV-2 gene fragment (Mem, Nuc, or ORF1a-Nsp1), and therefore an independent scrambled siRNA control was used for each line. The three grey “Scramble” columns in panels A–F correspond to these individual controls in Calu-3-Mem, Calu-3-Nuc, and Calu-3-Nsp1 reporter cells, respectively. **(G)** Comparative analysis of GFP silencing efficiency of each siRNA (18, 25, 27 and 30) across MC3, SM-102 and ALC-0315 LNP formulations at 25 nM, 50 nM and 100 nM. The graph summarizes the maximal gene-silencing efficiencies extracted from Fig. 2A–C for direct comparison among the three LNP formulations. All experiments were performed in triplicate. Data were presented as mean and standard deviation (SD). Statistical comparisons for panels A–F were performed using one-way ANOVA followed by Dunnett’s test to compare each siRNA treatment with its corresponding scrambled control. For comparative analyses among LNP formulations in panel G, one-way ANOVA followed by Tukey’s test was applied. * = *p* < 0.05, ** = *p* < 0.01, *** = *p* < 0.001, **** = *p* < 0.0001

To enable rapid, high throughput screening of the LNP-siRNA formulations in a biologically relevant cell, we generated lung epithelial, Calu-3 GFP reporter cell lines stably expressing SARS-CoV-2 Mem, Nuc or Nsp1. These GFP reporter cell lines were transfected with siRNAs 18, 25, 27 or 30 encapsulated by the various LNP formulations, which incorporated a Rhodamine (PE) fluorophore to enable quantification, and gene silencing was measured daily for 9 days using flow cytometry analysis of GFP suppression and LNP positive cells (Fig.S3-S5). Maximal GFP suppression was observed at day 5 post MC3 LNP-siRNA 18, 25, 27 and 30 (referred to as MC3 LNP-18, −25, −27, and −30; or simply MC3 18, 25, 27, and 30) treatments in Calu-3 GFP reporter cell lines, and significant silencing was maintained out to day 8 (Fig. 2A, S3). The SM-102- LNP-siRNA 18, 25, 27 and 30 (SM-102 LNP-18, −25, −27, and −30; or simply SM-102 18, 25, 27, and 30) and ALC-0315-based LNP-siRNA 18, 25, 27 and 30 formulations (ALC-0315 LNP-18, −25, −27, and −30; or simply ALC-0315 18, 25, 27, and 30) showed maximal GFP suppression at day 6, with significant silencing maintained out to day 9 in the SM-102-based formulation and day 8 for the ALC-0315-based formulation (Fig. 2B, 2C, S4, S5). All lead candidate siRNA (18, 25, 27 and 30) encapsulated in the three LNP formulations demonstrated significant GFP suppression. There was also a dose dependent effect observed in all LNP-siRNAs with increasing GFP suppression in the 50 and 100 nM treatments compared to the 25 nM treatment. At their respective optimal time points, MC3 LNP-18, −25, and −30 reduced target gene expression by approximately 14.0 %, 13.9 %, and 66.7 %, respectively, whereas SM-102 LNP-18, −25, and −30 achieved reductions of 20.8 %, 11.4 %, and 58.6 %. The corresponding ALC-0315 LNP-18, −25, and −30 reduced expression by 12.7 %, 12.7 %, and 45.9 %. Increasing the concentration of LNP-18, −25, and −30 to 50 nM further enhanced suppression to 20.6 %, 19.6 %, and 73.2 % for MC3; 30.0 %, 19.7 %, and 67.3 % for SM-102; and 20.6 %, 15.3 %, and 60.4 % for ALC-0315, demonstrating a clear dose-dependent trend across all formulations. For siRNA 27 and 30, the MC3 provided the greatest GFP suppression, followed by SM-102 and ALC-0315 (Fig. 2G).

Similarly, LNP-siRNA treated Calu-3 reporter cells showed the greatest percentage of Rhodamine fluorescence, indicating LNP positive cells, at day 5 for the MC3 LNP-siRNA (Fig. 2D), which remained high up to day 8 and confirms the significant GFP suppression observed at day 8 for MC3 LNP-18 and LNP-25 (Fig. S3), compared to siRNA controls. For MC3 LNP-27 and LNP-30, the monitoring was done until day 7. The SM-102- and ALC-0315-based LNP-siRNA 18, 25, 27 and 30 formulations also showed the greatest percentage of LNP positive cells at day 6 (Fig. 2E, 2F), and remained high until day 9 in the SM-102-based formulation and until day 8 in the ALC-0315-based formulation, which again confirms the significant GFP suppression reported at day 9 and 8, respectively, compared to siRNA controls (Fig.S4,5). This data demonstrates all three LNP formulations can deliver functional siRNA and induce significant gene silencing up to 8 days for the MC3- and ALC-0315-based formulations and up to 9 days for the SM-102-based formulation.

### Off-target effects of LNP-siRNA formulations

Since all three LNP formulations were able to deliver functional siRNA to Calu-3 cells, we next investigated the potential for induction of off-target effects in the type I interferon (IFN) pathway and expression of interferon stimulated genes (ISG). While type I interferons (IFNs) play a critical role in antiviral immunity [40], unintended activation of the type I IFN signalling pathway by siRNAs or their delivery nanocarriers can result in non-specific stimulation of innate immune responses. Such off-target immune activation is undesirable, as it may compromise therapeutic specificity, induce inflammation-related toxicity, and reduce overall treatment safety, particularly when delivered directly to the respiratory tract[36]. Therefore, assessing the induction of type I IFN responses by candidate siRNAs and associated nanocarriers, even at the in vitro stage, is essential to avoid off-target inflammatory responses as much as possible. This evaluation is crucial in the development of antiviral siRNA therapeutics, which ideally should avoid triggering innate immune recognition pathways[41, 42]. To assess this, mRNA expression levels of four interferon-stimulated genes (ISGs); IFIT1, OAS1, ISG20 and Viperin were measured in HeLa T4+ cells (non-pulmonary reference line) and Calu-3 cells (pulmonary epithelial model) at 3 days post-treatment for MC3, SM-102 and ALC-0315-based LNP formulations with lead candidate siRNA 18, 25, 27 and 30.

As shown in Fig.S6A-D, HeLa T4+ cells treated with the MC3 LNP formulation with siRNA 18, 25, 27 or 30 did not induce any measurable off-target effect in any of the four ISGs assessed. In contrast, among the ALC-0315 LNP-siRNA treatments, only siRNA 30 induced a significant increase in ISG20 expression (Fig.S6C). This was also the case for the SM-102-based LNP formulation, which induced significant ISG20 induction with siRNA 18, 25 and 30 treatments (Fig.S6C). Additionally, the SM-102-based LNP formulation also induced OAS1 expression with siRNA scrambled control, 27 and 30 treatments (Fig.S6B). Notably, the variability observed in Viperin expression in HeLa-T4⁺ mock samples likely reflect its very low basal transcriptional level in unstimulated cells, where small Ct fluctuations can amplify apparent fold-change differences after normalization (Fig.S6D). No off-target effect of ISGs IFIT1 or Viperin was observed in HeLa T4+ cells for any of the LNP formulations compared to the controls.

**Fig. 3:**
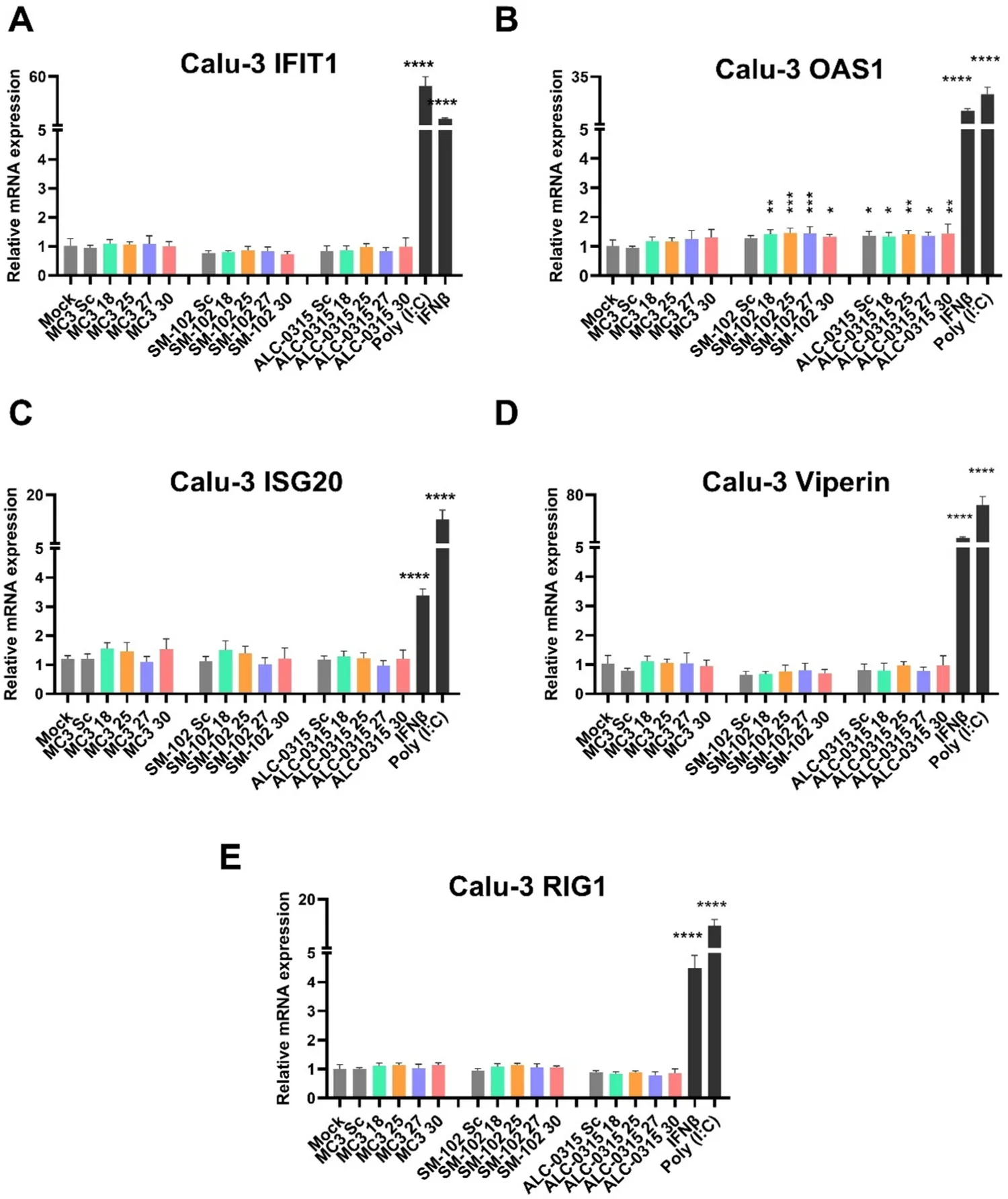
Assessment of off-target ISG effects of LNP-siRNAs in Calu-3 cells. Calu-3 cells (A-E) were seeded at 4 × 10^4^/well in 48-well plates and LNPs loaded with siRNA 18 (MC3 18, SM-102 18, and ALC-0315 18), siRNA 25 (MC3 25, SM-102 25, and ALC-0315 25), siRNA 27 (MC3 27, SM-102 27, and ALC-0315 27) or siRNA 30 (MC3 30, SM-102 30, and ALC-0315 30) at 50 nM were transfected 48 h after cell seeding. Post-treatment at 72 h, cells were lysed and collected for RNA extraction. RT-qPCR was performed for Calu-3 cells; (**A**) IFIT1, (**B**) OAS1, (**C**), Viperin, (**D**) ISG20, and (**E**) RIG1, and GAPDH was applied as the housekeeping gene. Each siRNA treatment was analysed together with its corresponding scrambled siRNA control for the same LNP formulation (MC3 Sc, SM-102 Sc, and ALC-0315 Sc). Relative quantification was determined (2.^−ΔΔCt^) and statistical comparisons were performed with Mock, excluding both positive Ctrls, IFN β and Poly (I:C), using One-Way ANOVA. Data were presented as mean and standard deviation (SD). Statistical significance was determined using one-way ANOVA followed by Dunnett’s test. * *p* < 0.05, ** = *p* < 0.01, *** = *p* < 0.001, **** = *p* < 0.0001

Assessment of off-target ISGs in Calu-3 cells demonstrated no induction of mRNA levels for IFIT1, ISG20, Viperin or RIG1 (Fig. 3A, C-E). In contrast, there was a significant induction of OAS1 in both the SM-102- and ALC-0315-based LNP-siRNA 18, 25, 27 and 30 treatments (Fig. 3B). None of the MC3 LNP-siRNA treatments induced OAS1 (Fig. 3B). This finding suggests that the specific lipid composition of SM-102 and ALC-0315 formulations may result in stronger endosomal membrane perturbation, thereby contributing to the observed interferon stimulation, although the precise upstream sensors remain incompletely defined.

Based on the findings of off-target ISG induction in both HeLa T4+ and Calu-3 cell lines, the SM-102-based LNP formulation was not explored in further investigations.

### Protective effect of LNP-siRNAs against SARS-CoV-2 in vitro

As the MC3 LNP formulation was identified to have the lowest immunogenicity, with no measurable off-target ISG induction, we next investigated the protective effect of the two most potent candidates, siRNAs 27 and 30, encapsulated in the MC3 formulation against SARS-CoV-2 infection. Vero E6 cells were treated with MC3 LNP-27 and MC3 LNP-30, either prior to infection with the Omicron BA.1 variant (pre-infection model) or after infection with the XBB.1.5 variant (post-infection model), and the viral load was measured based on nucleocapsid (NC) mRNA levels at day 5 post-infection. The Omicron BA.1 variant was used in the pre-infection model as it was the earliest Omicron strain available during initial experiments, allowing assessment of prophylactic protection. The more recent XBB.1.5 variant, known for its higher transmissibility and immune evasion, was applied in the post-infection model to evaluate therapeutic efficacy under a more stringent infection scenario.

**Fig. 4:**
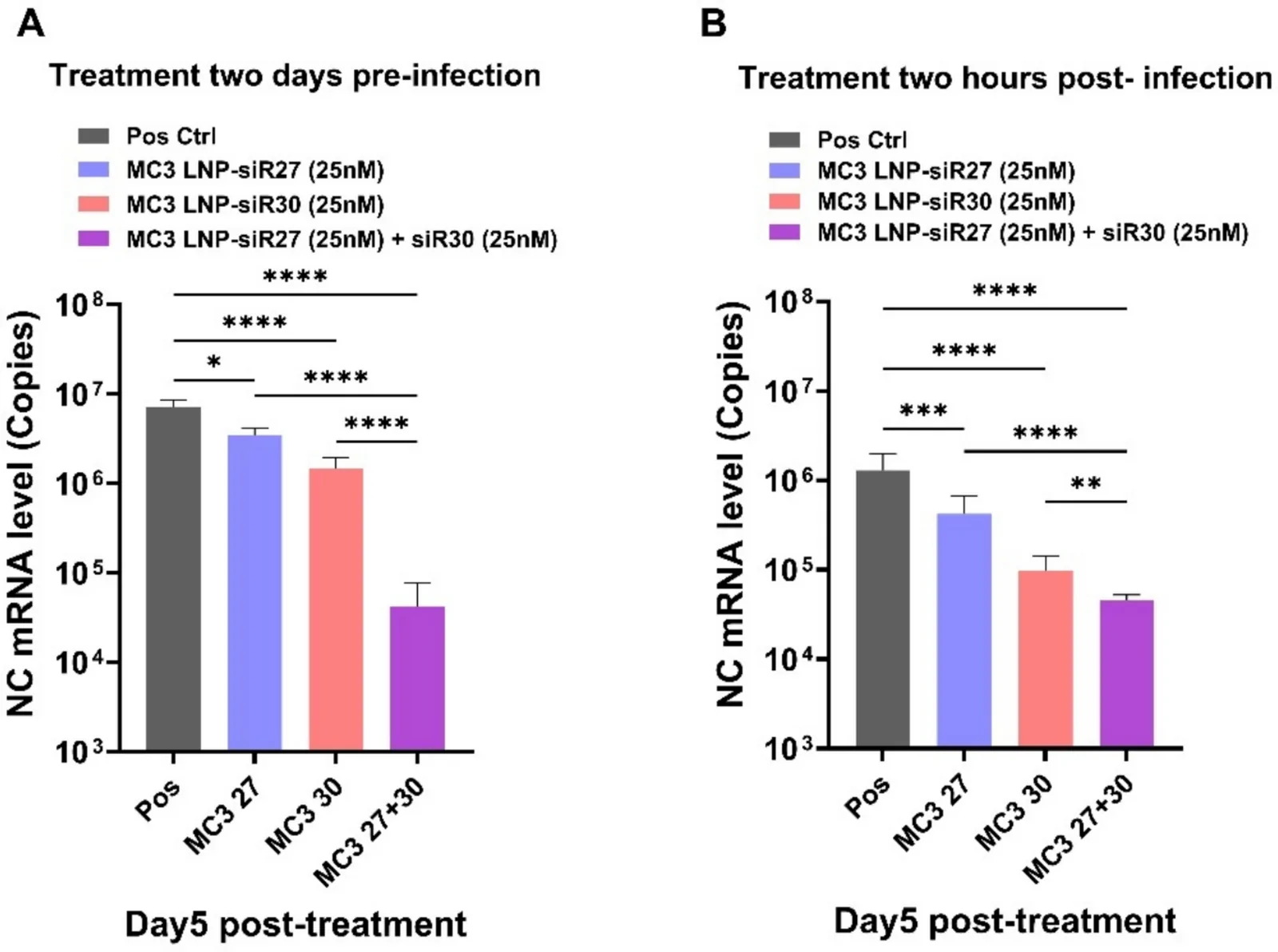
Protection of LNP-siRNAs on Vero E6 cells pre- and post-challenged with SARS-CoV-2 Omicron strains. (**A**) Viral load based on NC mRNA levels in Vero E6 cell supernatant following treatment with MC3-LNP-siRNA 27, 30, or 27 + 30 (MC3 LNP-27, MC3 LNP-30, MC3 LNP-27 + 30) at 25 nM two days pre- infection with SARS-CoV-2 Omicron BA.1 variant. Vero E6 cells were seeded 1.5 × 10^4^/well in 96-well plates. MC3 LNP-27 (25 nM) and MC3 LNP-30 (25 nM) were added 6 h after cell seeding. Post-treatment (48 h), cells were challenged with SARS-CoV-2 BA.1 with the MOI-0.05. Supernatant was collected Day 5 post-treatment. RNA extraction and RT-qPCR was performed to assess the NC mRNA level (log_10_ copies). (**B**) Viral load based on NC mRNA levels in Vero E6 cell supernatant following treatment with MC3 LNP-27, MC3 LNP-30, and MC3 LNP-27 + 30 (25 nM siRNA) 2 h post-infection with SARS-CoV-2 Omicron XBB.1.5 variant. Vero E6 cells were seeded 2 ×10^3^/well in 96-well plates, and SARS-CoV-2 XBB.1.5 infection was performed 6 h after cell seeding. MC3 LNP-27 and/or MC3 LNP-30 (25 nM) were then added 2 h post-infection (SARS-CoV-2 XBB.1.5 with MOI-0.5). Supernatant was collected Day 5 post-treatment. RNA extraction and RT-qPCR was performed to assess the NC mRNA level (Log_10_ copies). Data are presented as mean ± SD. Statistical comparisons were performed using one-way ANOVA followed by Tukey’s multiple comparisons test. * = *p* < 0.05, ** = *p* < 0.01, *** = *p* < 0.001, **** = *p* < 0.0001

The viral load was significantly decreased in Vero E6 cells treated with MC3 LNP-27 and MC3 LNP-30 pre-infection compared to the untreated infected group (Pos), with 0.31-log_10_ (p<0.05) and 0.69-log_10_ (p<0.0001) reductions, respectively. The combination LNP-siRNA 27+30 treatment induced the greatest decrease in NC mRNA levels, resulting in a 2.22-log_10_ reduction relative to the untreated infected control (*p*<0.0001, Fig. 4A).

To determine whether MC3 LNP-27 and/or MC3 LNP-30 were still able to suppress viral load when treatment commenced post-infection, we infected Vero E6 cells with the Omicron XBB1.5 variant then treated cells with MC3 LNP-27, 30 or 27+30 at 2 h post-infection. Similar to pre-infection treatment, MC3 LNP-27, 30 and the combination of 27+30 treatments all significantly reduced NC mRNA levels, with the combination MC3 LNP-27+30 inducing the greatest reduction of NC mRNA levels from 6.11 log_10_ copies to 4.66 log_10_ copies (*p*<0.0001 compared with Pos Ctrl, and p<0.01 compared with MC3 LNP-30 alone) (Fig. 4B). This data demonstrates MC3 LNP-27 and MC3 LNP-30 provide a protective antiviral effect with treatment pre- or post-infection.

### Assessment of gene silencing, off-target ISG and antiviral effect of LNP encapsulated Ome-AS456- modified siRNA

We next examined the MC3 and ALC-0315 LNP formulations with the Ome-AS456-modified siRNAs to determine whether any off-target ISG effect could be reduced, without compromising gene silencing and antiviral efficacy against SARS-CoV-2 infection.

LNP encapsulated OmeAS456-modified-siRNA 30, formulated as either MC3 LNP-siRNA 30mod (MC3-30mod) or ALC-0315 LNP-siRNA 30mod (ALC-0315-30mod), were generated and validated using the Calu-3 Nsp1-GFP reporter cell line. As shown in Fig.S7A, all three concentrations (25, 50 and 100 nM) of both MC3-30mod and ALC-0315-30mod significantly down-regulated GFP expression from day 3 to day 10 post-treatment, with the MC3-30mod achieving significantly greater suppression compared to the ALC-0315-30mod. Additionally, GFP suppression in MC3-30mod and ALC-0315-30mod was highest at both day 6 and 7 for all three concentrations. Similarly, the percentage of LNP positive cells was >90% from day 3 to 7 for MC3-30mod and MC3-Scrambled control for all concentrations (Fig.S7B).

**Fig. 5:**
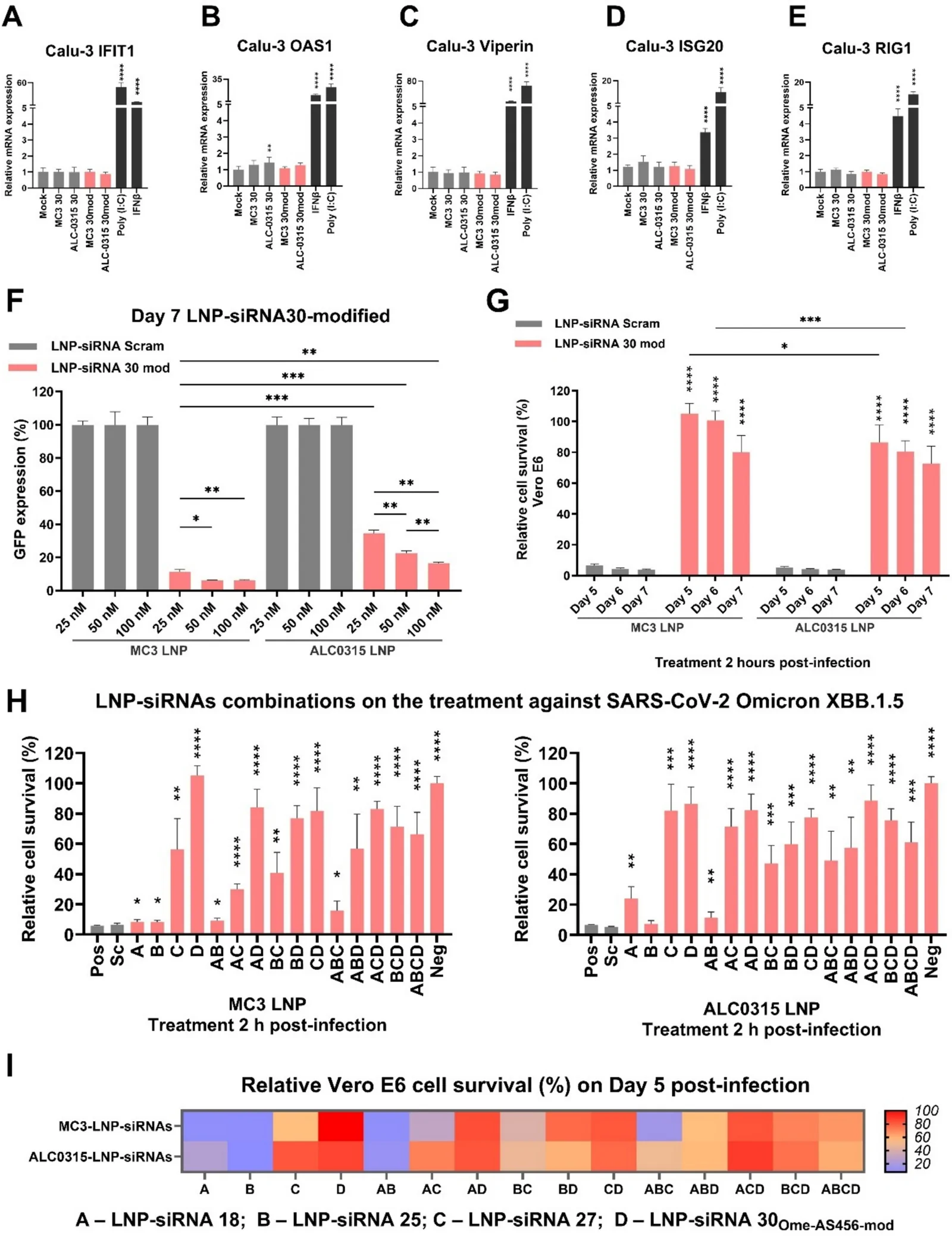
Assessment of ISG activation, gene silencing, and antiviral effects of single and multiplexed LNP-modified siRNAs infected with SARS-CoV-2. Calu-3 cells were seeded 4 × 10^4^/well in 48-well plates and treated with MC3 LNP-OmeAS456 modified-siRNA 30 (MC3-30mod) and ALC-0315-LNP-modified-siRNA 30 (ALC-0315-30mod) at 50 nM 48 h after cell seeding. 72 h post-treatment, cells were lysed and collected for RNA extraction. RT-qPCR was performed for **(A)** IFIT1, **(B)** OAS1, **(C)**, Viperin, **(D)** ISG20, **(E)** RIG1 and GAPDH was used as a housekeeping gene. Relative quantification was then determined (2^−ΔΔ*Ct*^) and statistical comparisons were performed with mock, excluding both positive Ctrls, IFN β and Poly (I:C) using One-Way ANOVA followed by Dunnett’s test. **(F)** Maximal gene silencing at Day 7 in Calu-3 Nsp1-GFP reporter cells treated with 25, 50, or 100 nM LNP-siRNA 30, comparing MC3—versus ALC-0315-based formulations. GFP expression was analyzed by flow cytometry; data were normalized to LNP-siRNA-Scramble. One-Way ANOVA followed by Tukey’s test was applied. **(G)** Cell survival assay of VeroE6 cells treated with 25 nM of MC3-30mod and ALC-0315-30mod at 2 h post-infection with SARS-CoV-2 XBB.1.5. NucBlue staining and IN Cell Analyzer were used to image live cells from Day 5 to 7 post-infection. Two-Way ANOVA followed by Šídák’s test was applied. **(H**) Therapeutic effects of LNP-siRNA combinations against SARS-CoV-2 Omicron XBB.1.5 infection. Vero E6 cells were seeded 2×10^3^/well in 96-well plates, and SARS-CoV-2 XBB.1.5 infection (MOI-0.5) was performed 6 h after cell seeding. Combination of MC3-LNP-siRNAs (A- MC3 LNP-18; B- MC3 LNP-25; C- MC3 LNP-27; D- MC3 LNP-30-OmeAS456-mod) and combinations of ALC-0315-LNP-siRNAs (A- ALC-0315 LNP-18; B- ALC-0315 LNP-25; C- ALC-0315 LNP-27; D- ALC-0315 LNP-30-OmeAS456-mod) were then added 2 h post-infection. Each LNP-siRNA in the combination was added in equal amounts, with a total concentration of 25 nM. NucBlue was added to stain live cells 5 days post-infection, and cells were imaged to quantitate the relative cell survival % using the IN Cell Analyzer at day 5 post-infection/treatment. All live cell percentages were normalized with the negative control (neither LNP-siRNAs or SARS-CoV-2 were added). Statistical comparisons were performed with the infected siRNA-Scramble group using One-Way ANOVA followed by Dunnett’s test. **(I)** Heat-map of the therapeutic effect of LNP-siRNA combinations against SARS-CoV-2 Omicron XBB.1.5 infection. Three colours, blue (< 20%), orange (20–80%) and red (> 80%) were used to evaluate the Vero E6 cell survival (%) of combination. Data were presented as mean and standard deviation (SD). All experiments included six replicates. * = *p* < 0.05, ** = *p* < 0.01, *** = *p* < 0.001, **** = *p* < 0.0001

Assessment of off-target ISG effects demonstrated there was no induction of IFIT1, ISG20, Viperin or RIG1 in either MC3- or ALC-0315-based LNP formulation with siRNA 30 or siRNA 30 modified (Fig. 5A, C-E). Surprisingly, the significant induction of OAS1 observed for the ALC-0315 LNP-30 treatment was decreased to control levels when siRNA 30 was chemically modified with 2’-Ome on the antisense strand at positions 4, 5 and 6 (Fig. 5B). This suggests the 2’-Ome modification is beneficial for reducing ISG off-target effects.

Screening of GFP suppression in the Calu-3 Nsp1-GFP reporter cell line showed maximal silencing at day 7 post-treatment with both MC3-30mod and ALC-0315-30mod at all concentrations (25, 50 and 100 nM) (Fig. 5F). Interestingly, even at the lowest dose of 25 nM, MC3-30mod achieved significantly stronger gene silencing than ALC-0315-30mod at 25 nM, 50 nM, and 100 nM (Fig.5F). Both MC3-30mod and ALC-0315-30mod at 25nM two hours post-infection was observed significant protection of VeroE6 cells against SARS-CoV-2 Omicron XBB.1.5 from Day5 to Day 7 post-infection. Similarly, on Days 5 and 6 post-infection, treatment with MC3-30mod resulted in significantly higher cell survival compared to ALC-0315-30mod at equivalent dosing. **(**Fig. 5G**)**

To investigate potential combinatorial effects, various combinations of LNP-siRNAs were evaluated for their ability to protect Vero E6 cells against SARS-CoV-2 Omicron XBB.1.5 infection. Among the MC3-based formulations, combinations such as AD (MC3 18+30), CD (MC3 27+30), ACD (MC3 18+27+30), and ABCD (MC3 18+25+27+30) exhibited protective effects, each maintaining over 80% relative cell survival following infection. Although the apparent protection achieved by the single 25 nM MC3-30mod treatment was higher, this likely reflects the fact that, under the same total siRNA concentration (25 nM), siRNA 30 constituted 100 % of the dose in the single treatment but only 50 % (e.g. MC3 27+30) or 25 % (e.g. MC3 18+25+27+30) in the combination groups.

A similar trend was observed with ALC-0315 LNP-siRNAs, where the inclusion of siRNA 30 enhanced antiviral efficacy. These LNP-siRNA combinations not only demonstrated robust therapeutic effects but may also offer a strategic advantage in combating potential future viral mutations by simultaneously targeting multiple conserved genomic regions **(**Fig. 5H**)**. Given its robust gene silencing, reduced off-target ISG activation, and stronger antiviral efficacy relative to the ALC-0315-based formulation, further investigation focused on evaluating the therapeutic potential of MC3 LNP-siRNAs at an initial dose of 25 nM.

### Post-infection therapeutic efficacy of MC3 LNP-siRNAs in Vero E6 and Calu-3 cells

**Fig. 6:**
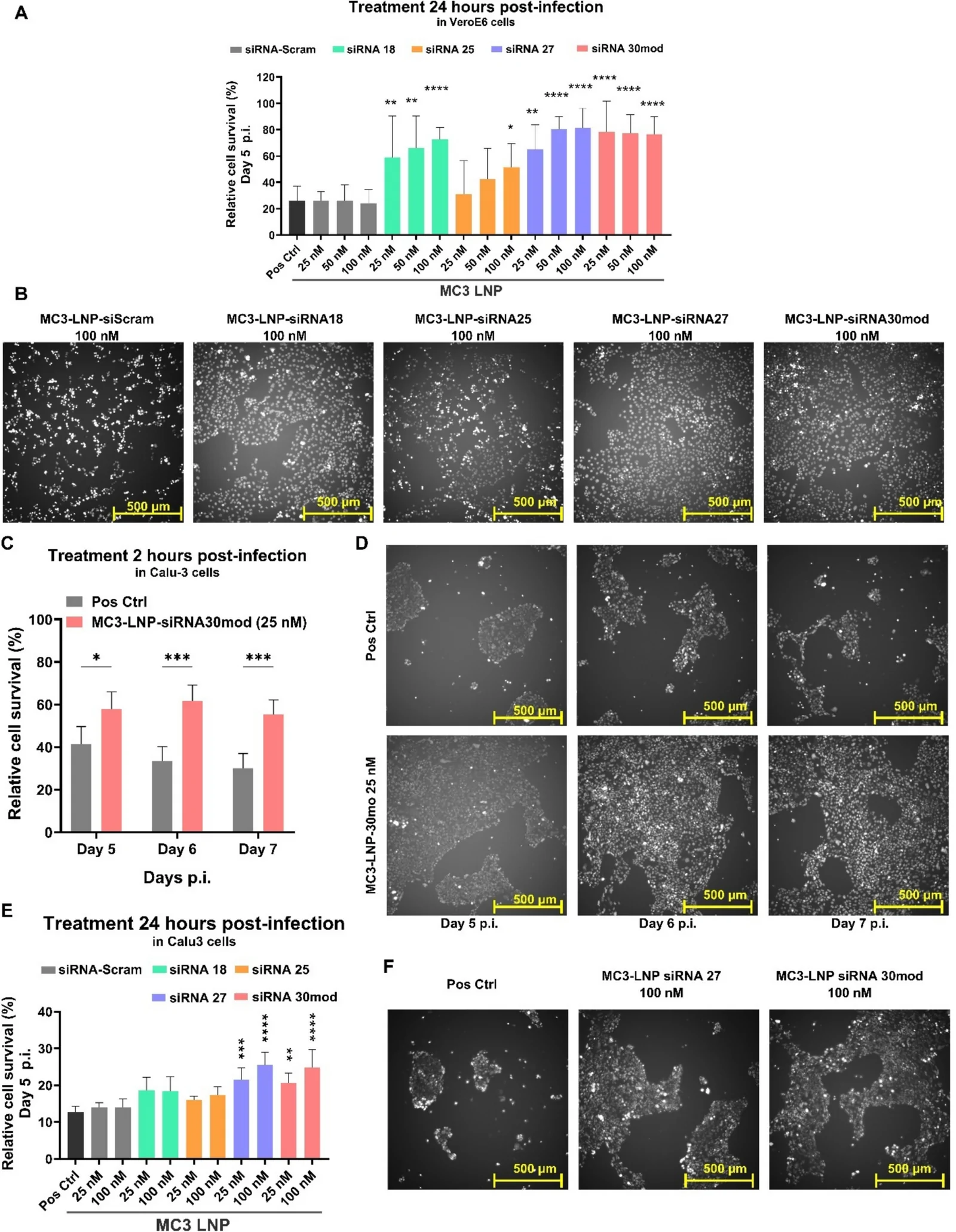
Antiviral efficacy of LNP-modified siRNAs 2 h and 24 h post-infection of SARS-CoV-2. (**A**) Protective effects of MC3 LNP-siRNAs administered 24 h post-infection in Vero E6 cells infected with SARS-CoV-2 XBB.1.5 (MOI—0.5). Cell survival assay was assessed via NucBlue staining and IN Cell Analyzer on Day 5. (**B**) IN Cell Analyzer images of NucBlue™ stained Vero E6 cells treated with 100 nM MC3-LNP-siRNAs 5 days following Omicron XBB.1.5 infection. (**C**) Cell survival assay of Calu-3 cells treated with MC3 LNP-siRNA 30mod 2 h post-infection with Omicron XBB.1.5. (**D**) IN Cell Analyzer images of NucBlue™ stained Calu-3 cells treated with MC3 LNP-siRNAs 5 to 7 days following Omicron XBB.1.5 infection. (**E**) Cell survival assay of Calu-3 cells treated with MC3 LNP-siRNAs on Day 5 post-infection with Omicron XBB.1.5. (**F**) IN Cell Analyzer images of NucBlue™ stained Calu-3 cells treated with MC3 LNP-siRNAs 5 days following Omicron XBB.1.5 infection. All live cell percentages were normalized with the negative control. All experiments were performed with six replicates. Data were presented as mean and standard deviation (SD). Statistical comparisons were performed with infected siRNA-Scramble group with the same siRNA concentration using One-Way ANOVA followed by Dunnett’s test (panels A and E). Two-way ANOVA was used to assess the effects of treatment and time, followed by Šídák’s test to compare treatment groups at each time point (panels C). * = *p* < 0.05, ** = *p* < 0.01, *** = *p* < 0.001, **** = *p* < 0.0001

The therapeutic efficacy of all MC3 LNP-siRNAs was examined in both Vero E6 and Calu-3 cells, with treatment delayed until 24 h post-infection. All four MC3 LNP-siRNA 18, 25, 27, and 30mod at 100 nM still protected Vero E6 cells against virus infection for at least 5 days (Fig. 6A-6B), and LNP-siRNA 18, 27 and 30mod also protected at lower concentrations of 25 and 50 nM (Fig. 6A). Protection was also observed at day 6 and 7 post-infection for LNP-siRNAs 18, 27 and 30mod (Fig.S9). In Calu-3 cells, treatment with MC3 LNP-siRNA 30mod at 25 nM, administered two hours post-infection, resulted in significant protection for at least seven days compared to the control (Fig.6C-6D). Interestingly, when treatment was delayed until 24 hours post-infection, Calu-3 cells showed maximum protection with the LNP-siRNA 27 and 30mod treatments at 25 and 100, while there was no clear virus suppression with siRNAs 18 and 25 (Fig.6E-6F). The protective effect of MC3 LNP-siRNAs 27 and MC3 LNP-30mod was also observed at days 6 and 7 post-infection (Fig.S9). Combined this data demonstrates the MC3 LNP-siRNA 27 and 30mod treatment provide greater antiviral effect and the significant silencing of virus infection with treatment 24 h post-infection in vitro shows potential for a post-exposure prophylaxis treatment in vivo.

### Biosafety assessment of single doses of LNP-siRNAs in vivo

**Fig. 7:**
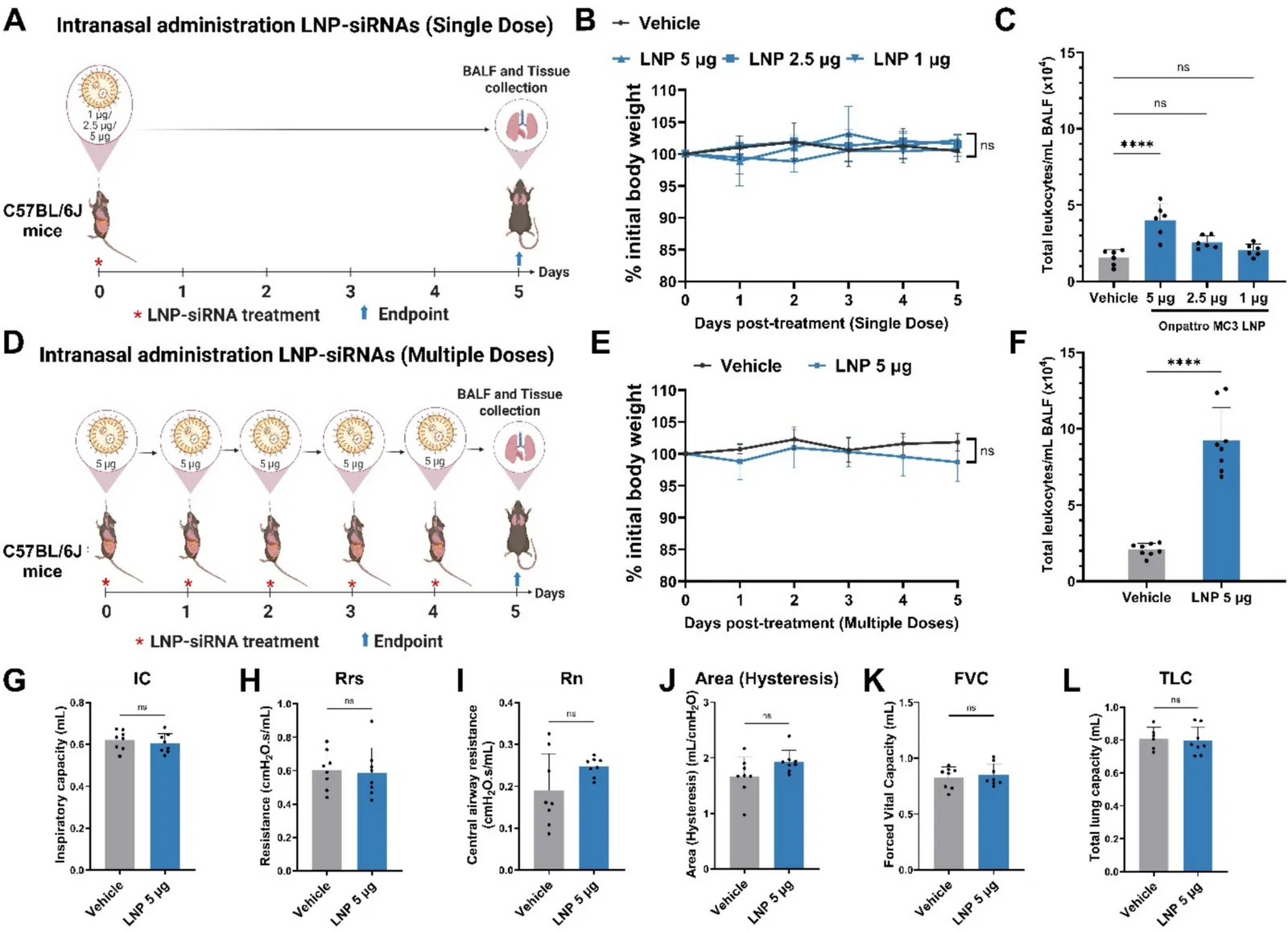
In Vivo toxicity testing with single and multiple doses of intranasally delivered LNP-siRNA in C57BL/6 J mice. (**A**) Single dose of intranasally delivered LNP-siRNA in C57BL/6 J mice. Four groups of C57BL/6 J mice with 6 mice in each group were inhaling MC3-LNP-siGFP with 1ug, 2.5ug, and 5ug siRNA per mouse, with just vehicle (PBS) as negative control. Inhalation of LNP-siRNAs was performed on Day 0, and harvest on day 5. (**B**) Body weight of mice in each group with single dose. The body weight of each mouse was weighed and recorded daily. (**C**) Lymphocytes in the bronchoalveolar lavage fluid (BALF) of mice in each group was tested by cell counting. (**D**) Multiple doses of intranasally delivered LNP-siRNA with 5ug per mouse. Two groups of C57BL/6j mice with 8 mice in each group were inhaling LNP-siGFP with 5ug siRNA per mouse, or with PBS as vehicle control. The treatment was performed daily for five days from Day 0 to Day 5, with harvest on Day 5. (**E**) Body weight of mice in each group with multiple doses. The body weight of each mouse was weighed and recorded daily. (**F**) Lymphocytes in the BALF of mice in each group. (**G**) The Inspiratory capacity (IC), (**H**) resistance of respiratory system (Rrs), (**I**) resistance of central airways (Rn), (**J**) Hysteresis Area, (**K**) Forced Vital capacity (FVC), and (**L**) Total lung capacity (TLC) of mice in each group was tested. Statistical analyses were performed using Two-Way ANOVA followed by Šídák’s test for body weight measurements (panels B and E) and One-Way ANOVA followed by Dunnett’s test for panels C. Student’s t-test was applied for two independent group comparisons (panels F–L). * = *p* < 0.05, ** = *p* < 0.01, *** = *p* < 0.001, **** = *p* < 0.0001

Onpattro, developed by Alnylam, employs MC3-based lipid nanoparticles for siRNA delivery via intravenous administration. However, given that the present study explores respiratory delivery, it is critical to assess whether the same LNP-siRNA formulation induces any adverse effects when delivered via the respiratory tract. We therefore examined the respiratory delivery of the MC3 LNP-siRNA formulation and identified the maximum tolerated dose (MTD) in vivo. C57BL/6J mice were treated with a single dose of 1 μg (≈ 0.05 mg/kg), 2.5 μg (≈ 0.125 mg/kg), or 5 μg (≈ 0.25 mg/kg) of LNP-siRNA targeting GFP (MC3 LNP-siGFP) [43] via intranasal instillation (Fig. 7A). Mice maintained stable body weight over 5 days following single-dose LNP-siRNA administration, with all three treatment groups (1 µg, 2.5 µg, 5 µg) showing no statistically significant deviation from the vehicle group (Fig.7B). Single-dose administration of 1 μg or 2.5 μg did not result in increased leukocyte infiltration in BALF (Fig. 7C). In contrast, the 5 μg dose significantly elevated total BALF leukocytes from 1.66 × 10^4^/mL to 4.01 × 10^4^/mL, supporting the presence of an inflammatory response in the lung. Given that intranasal instillation primarily deposits the administered droplets into the lower respiratory tract when applied at controlled volume and position, the observed pulmonary immune response indicates direct lung exposure rather than systemic absorption. Future biodistribution studies with fluorescently labelled siRNAs are required to confirm pulmonary localisation and to quantify potential systemic distribution in peripheral organs.

### Biosafety assessment of multiple doses of LNP-siRNAs in vivo

To assess the effects of repeated respiratory dosing, 5 μg of MC3 LNP-siGFP (or vehicle; PBS) was administered intranasally once daily for five consecutive days (n = 8 per group) (Fig. 7D). As shown in Fig.7E, body weight remained stable over the course of treatment, with no statistically significant loss observed. BALF analysis revealed a further increase in inflammatory cell infiltration following repeated dosing, with leukocyte counts rising from 2.07 × 10^4^/mL to 9.22 × 10^4^/mL (Fig. 7F). Comprehensive pulmonary function tests—including inspiratory capacity (IC), total respiratory resistance (Rrs), central airway resistance (Rn), hysteresis area, forced vital capacity (FVC), and total lung capacity (TLC)—showed no significant differences between the LNP-siRNA and vehicle-treated groups (Fig. 7G–7L), indicating that repeated intranasal dosing did not induce measurable functional impairment over 5 days. However, the concurrent increase in BALF leukocyte counts suggests a localized inflammatory response, consistent with mild pulmonary irritation rather than systemic toxicity.

## Discussion

Building upon our previous identification of four potent anti-COVID siRNA candidates, this study systematically evaluated their delivery efficiency, off-target immune responses, and post-infection therapeutic potential when encapsulated in various lipid nanoparticle (LNP) formulations [44]. A systematic comparison was conducted for LNP-siRNA formulations derived from three clinically approved platforms—Alnylam (Onpattro) [45], Moderna [46–49], and Pfizer [50–52]—with siRNA chemical modifications to minimize off-target responses. This study is the first to demonstrate significant antiviral efficacy of clinically relevant LNP-siRNA therapeutics administered 24 hours post-SARS-CoV-2 infection in vitro, as previous studies have not reported antiviral activity with this time window using LNP-delivered siRNAs [53, 54]. This post-exposure intervention model marks a significant advance in the delivery of siRNA therapeutics, highlighting the potential of LNP-siRNA not only for treatment, but also for post-exposure prophylaxis.

Several studies have reported that LNP delivery platforms can effectively facilitate the delivery of siRNAs following both intravenous injection [55, 56] and intranasal administration [57–59] for therapeutics against SARS-CoV-2 infection. MC3-based formulation, as the delivery platform for the first FDA-approved siRNA therapeutic, served as our proof-of-concept vehicle. When tested in 293 T GFP reporter cells fused SARS-CoV-2 targeting regions, it was found that MC3 LNP-siRNAs demonstrated efficient and sustained delivery, with 25 nM doses achieving ~50% of gene silencing. (Fig. 1) Building on this, we extended our analysis to physiologically relevant pulmonary epithelial models by generating sub-bronchial epithelial Calu-3 reporter cells stably expressing SARS-CoV-2 Membrane, Nucleocapsid, and Nsp1 targets (Fig.S1), a reliable and sensitive preclinical model for investigating drug delivery in the upper respiratory tract [60, 61], and for studying various human respiratory diseases, especially SARS-CoV-2 infection [25, 62–64]. Three LNP formulations based on FDA-approved RNA-based therapeutics— MC3 (Alnylam), SM-102 (Moderna), and ALC-0315 (Pfizer) —were generated and systematically compared in terms of their ability to deliver functional siRNAs to sub-bronchial epithelial Calu-3 reporter cells for functional silencing. Among the three, MC3 formulated LNPs consistently demonstrated superior and stable cellular uptake compared to SM-102 and ALC-0315 formulations, especially at lower concentrations (25 nM). Similarly, MC3 LNP-siRNAs also achieved the highest level of GFP suppression at various concentrations tested (25 nM, 50 nM, and 100 nM), highlighting the relationship between efficient cellular uptake and potent gene silencing (Fig.2, Fig.S3-S5). Notably, the SM-102 and ALC-0315 formulations also achieved robust siRNA delivery and silencing with slightly delayed peak activity. These findings revealed that all three clinically approved LNP platforms are capable of facilitating efficient siRNA delivery to respiratory epithelial cells, supporting their potential use in pulmonary RNAi therapeutics. Given the clinical relevance of respiratory delivery, the next phase of our investigation focused on evaluating whether these LNP-siRNAs induce off-target immunostimulatory effects, particularly the activation of interferon-stimulated genes (ISGs).

While siRNAs 18 and 25 had previously shown no ISG-related off-target effects, and siRNAs 27 and 30 with 2'-O-methyl modifications at antisense positions 4, 5, and 6 as distinct from their unmodified version, did not induce ISGs expression, the potential ISGs immunostimulatory impact of the LNP carrier itself required further evaluation—especially given the potential for respiratory delivery to elicit inflammation in the upper or lower airways [65]. LNP components are known to induce immunogenicity, such as the ionizable cationic lipid component in the SM-102 LNP formulation inducing interleukin (IL)−1β release in human peripheral blood cells (PBMCs) [66] and anti-PEG antibodies inducing activation via the complement pathway[67, 68].

Given the clinical importance of minimizing ISG-related immunogenicity, the potential activation of ISGs by these LNP-siRNAs was subsequently assessed. Our investigation of off-target interferon responses demonstrated the SM-102, followed by the ALC-0315 LNP formulations both induced some level of ISG expression, while the MC3 LNP formulation did not. This inflammatory effect was observed in two different cell types, indicating the induced ISG expression was not cell type specific (Fig. 3). The main difference in the three LNP formulations is their unique ionizable cationic lipid, suggesting that the SM-102 and ALC-0315 may be relatively more immunogenic than the MC3, which is consistent with their primary use case, the former being used in vaccination the latter for delivery of siRNA therapeutics by IV administration. The superior safety profile of MC3 LNPs likely arises from physicochemical characteristics of its ionisable lipid (pKa ≈ 6.4), which maintains a near-neutral charge at physiological pH, thereby reducing nonspecific protein interactions and pattern recognition receptor activation, while becoming protonated in acidic endosomes to promote efficient siRNA release [69, 70]. Furthermore, lipid composition and biodistribution remain critical determinants of the in vivo translation and safety of ionisable lipid nanoparticles [29].

While transient ISG activation is an integral component of antiviral host defence and can, in some contexts, act as a beneficial adjuvant, the respiratory epithelium has limited tolerance for repeated particulate exposure, and intranasally delivered LNPs have been associated with BALF leukocytes and local inflammatory responses in vivo [71]. In view of the intended intranasal route, we therefore adopted a conservative selection strategy that prioritised formulations capable of robust, sequence-specific RNAi while minimising measurable ISG induction in vitro, with the aim of reducing the risk of excessive mucosal inflammation upon translation in vivo. Within this framework, the MC3 LNP platform was particularly attractive, as it combined the most favourable ISG profile with efficient and durable target knockdown and antiviral activity in Calu-3 cells, and superior performance of MC3-30mod compared to ALC-0315-30mod at equivalent dosing. Nevertheless, future studies may explore whether carefully controlled ISG-inducing formulations could offer added benefit in specific therapeutic contexts. Moreover, both single and combined LNP-siRNAs treatments pre-infection significantly reduced viral nucleocapsid mRNA levels and preserved cell viability, and treatments post-infection also resulted in robust suppression of viral gene expression and protection from virus-induced cell death, which supports the suitability of MC3 LNP formulation as a safe and effective delivery vehicle for siRNA therapeutics for both prophylactic and post-exposure treatment (Fig. 4). However, because infectious virion output was not directly quantified by plaque assay, these data should be interpreted as evidence of strong inhibition of viral gene expression and a consequent protection of cell viability, rather than a definitive demonstration of reduced infectious viral load. These observations may point toward a structure–activity relationship in which the physicochemical features of MC3, particularly its pKa and tail architecture, could contribute to a more favourable balance between endosomal escape and innate immune activation relative to SM‑102 and ALC‑0315. This potential advantage might help explain why MC3 was selected as the lead formulation in this study. Taken together, the MC3 LNP formulation appears to possess properties that could make it a clinically suitable delivery vehicle for siRNA therapeutics targeting cells in the respiratory tract.

Building upon the limited off-target effect of the MC3 and ALC-0315 LNP formulations in this study, we further evaluated whether LNP encapsulated chemically modified siRNA 30 (LNP 30mod) could achieve potent antiviral effects 2 hours post-infection while minimizing off-target ISG activation. The modification of 2'-O-methyl at positions 4, 5, and 6 of the antisense strand effectively suppressed the previously observed ISG induction, particularly OAS1, in both MC3- and ALC-0315 LNP-30 (Fig. 5A-5E). Notably,** t**he MC3 30mod demonstrated significant superior gene silencing at 25 nM compared to ALC-0315 30mod at concentrations up to 100 nM (Fig. 5A-5F**, Fig S7**). For antiviral efficacy, MC3-30mod also significantly enhanced cell survival rates relative to the ALC-0315-30mod administered two hours post-infection, indicating stronger protection of MC3-30mod against Omicron XBB.1.5 variant infection (Fig. 5G). These highlight the utility of strategic chemical modification in enhancing immunological compatibility without compromising antiviral efficacy. Unlike monoclonal antibodies or traditional antivirals that require frequent updates [72–75], a two-siRNA combination offers greater resilience to viral escape mutations[76]. In this study, dual, triple, and even quadruple combinations of LNP-siRNAs administered post-infection with a total concentration of 25 nM provided robust antiviral activity without the need for sequence adjustments, supporting their resilience against viral evolution (Fig. 5H, Fig. S8). These findings further support the MC3 LNP platform as a promising intranasal delivery system for siRNA therapeutics employing multiplexed strategies, and underscore its suitability for continued evaluation of extended post-exposure antiviral efficacy.

Based on the above findings, the MC3 LNP platform was selected for further evaluation at an extended treatment window, 24 hours post-infection, in both Vero E6 and human epithelial Calu-3 cells. Here, all four candidate siRNAs were individually encapsulated, with siRNAs 18, 25, and 27 used in their unmodified form due to the absence of detectable ISG-related off-target effects in earlier assessments, while siRNA 30 was chemically modified with 2′-O-methyl substitutions to mitigate its previously observed immunogenicity**.** Of the four tested siRNAs, MC3 LNP-30mod and MC3 LNP-27 demonstrated the best antiviral activity at 25 and 100 nM, with significant protection maintained for up to seven days post-infection in both VeroE6 and Calu-3 cells (Fig. 6). Interestingly, siRNA 25 induced strong GFP suppression but conferred limited antiviral protection, whereas siRNA 27 exhibited modest knockdown in reporter cells yet provided robust antiviral activity, despite full sequence complementarity (Fig. 2A, 6A). This apparent discrepancy likely reflects differences between the reporter constructs and the native viral RNA transcripts in infected cells. It is also possible that the relevant viral transcript can be efficiently targeted and/or degraded by siRNA 27 only in the context of active viral replication, whereas the overexpressed reporter mRNA does not fully recapitulate this architecture or turnover. These results highlight the limitations of relying solely on sequence complementarity or overexpression models to predict antiviral efficacy. Functional validation using live-virus challenge assays remains the in vitro gold standard for the accurate identification of potential siRNA therapeutics.

To translate the promising in vitro performance of MC3 LNP-siRNA formulations into clinically viable respiratory therapeutics, in vivo biosafety must be rigorously assessed. The intranasal doses used for the in vivo biosafety assessment (1–5 µg siRNA per mouse) were not intended as a direct stoichiometric translation of the effective in vitro concentrations (25–100 nM). In vitro siRNA concentrations reflect local extracellular exposure under controlled culture conditions, whereas intranasal administration in vivo involves complex variables including airway deposition, mucociliary clearance, epithelial uptake efficiency, and formulation-dependent distribution. Accordingly, the selected dose range was informed by previously reported intranasal siRNA and LNP–siRNA studies rather than by direct concentration matching. For example, intranasal delivery of free or conjugated siRNAs has commonly been performed in the 30–50 µg per mouse range without overt toxicity [33, 77], while DOTAP- and dmLNP-formulated siRNAs have been administered either intravenously or intranasally at 1 mg/kg doses depending on formulation and route [57]. In this study, the 1–5 µg MC3 LNP-siRNA (0.05–0.25 mg/kg) was selected as a conservative intranasal dosing window to assess pulmonary tolerability of MC3 LNP–siRNAs prior to antiviral efficacy studies, with repeated daily dosing over five consecutive days corresponding to a cumulative exposure of approximately 0.25–1.25 mg/kg.

Here, we observed that intranasal delivery of MC3 LNP-siRNA at lower doses (1–2.5 μg) was well tolerated in C57BL/6J mice, as reflected by stable leukocyte counts in BALF and unchanged body weight. However, a single higher dose of 5 μg triggered a mild but statistically significant increase in pulmonary leukocytes, indicative of localized inflammatory activation (Fig. 7A-7C). This effect was further amplified with repeated daily dosing at 5 μg, where BALF leukocyte infiltration nearly quadrupled, suggesting a dose- and frequency-dependent immune response in the lung. Despite this, no detectable impairment in overall pulmonary function was observed, as key respiratory mechanics, including total lung capacity, inspiratory capacity, and airway resistance, remained comparable to vehicle-treated controls (Fig. 7D-7L). In the repeated-dose study, the highest single-dose level (5 μg; 0.25 mg/kg) was selected as a stringent local‑tolerance scenario, rather than as a proposed therapeutic regimen, in order to explore the upper bound of acceptable pulmonary inflammation. Lower doses (1–2.5 μg), which did not increase BALF leukocytes after a single instillation, were not repeated here but will be prioritised in future intranasal efficacy and dose-optimisation studies, as well as optimising the formulation. These results demonstrate that while transient lung inflammation can be induced by higher or repeated intranasal LNP exposure, such responses may be largely self-limited and do not compromise essential lung physiology, at least in the 6 day timeframe investigated. Nonetheless, given previous observations that elevated cationic lipid content contributes to enhanced immunogenicity and reduced safety [78–80], this emphasizes the need for further optimization of siRNA-LNP formulation or alternative dosing regimens to reduce local immune stimulation without sacrificing delivery efficiency.

One limitation of the current study is that the in vivo component focused on tolerability and pulmonary safety following intranasal administration in mice and did not include viral challenge experiments or direct measurements of infectious viral burden. Accordingly, conclusions regarding antiviral efficacy are presently restricted to in vitro models, including human pulmonary epithelial Calu-3 cells. As establishing respiratory safety and dose tolerability is a critical prerequisite for advancing intranasally delivered LNP–siRNA therapeutics into infected animal models, future studies will be required to evaluate MC3 LNP-siRNA formulations on the reduction of viral replication and infectious titres in appropriate SARS-CoV-2 challenge models, such as hACE2 mice [81], to fully define in vivo antiviral efficacy and translational potential. In parallel, dedicated pharmacokinetic and biodistribution studies will be needed to characterise the clearance of LNP-encapsulated siRNAs from the respiratory tract and secondary organs following intranasal administration. Moreover, assessing the physicochemical stability and mucus interactions of these LNP formulations under conditions that more closely mimic the airway environment could help improve understanding of formulation behaviour during intranasal delivery and optimise delivery efficiency within the respiratory tract.

A further limitation of the present in vivo work is the use of intranasal instillation rather than aerosolised nebulisation. While instillation is widely used as an initial screening approach and allows precise control of the nominal dose, it does not fully recapitulate the more homogeneous and physiologically relevant deposition patterns achievable with nebulisers. Therefore, future work will validate the efficacy of LNP-siRNAs via nebulization to strengthen the clinical relevance of this promising platform. Longer-term intranasal dosing studies will also be conducted to determine whether the observed inflammation is sufficient to cause alterations in pulmonary function associated with chronic inflammatory disease. In addition, further evaluation of atomizing delivery devices may provide further potential in mucosal tolerability and antiviral performance. From a broader perspective, these findings highlight the importance of developing efficient respiratory delivery systems—such as peptide dendrimer[82], cell-penetrating peptides[83], or next-generation lipid nanoparticles—to enhance siRNA stability and therapeutic efficacy with reduced systemic side effects in vivo [84]. Development of alternative delivery vectors and optimized atomization devices will be essential for translating these findings into effective clinical interventions.

In conclusion, we developed a comprehensive, novel and clinically relevant LNP-siRNA therapeutic platform for SARS-CoV-2 infection by integrating siRNA efficacy verification in vitro, LNP delivery, and immune-silencing chemical modifications. Importantly, the platform was shown to maintain antiviral efficacy even when siRNA-LNPs were administered 24 h post-infection. This extended treatment window expands the potential therapeutic applicability of RNAi-based antivirals and supports their use in post-exposure scenarios, where early intervention may not always be feasible. Future investigations will focus on optimizing and validating the safe dosage and therapeutic efficacy of this platform in animal models, particularly through intranasal delivery. Taken together, the ability of our siRNA-LNP platform to retain efficacy without the need for variant-specific updates, highlights its promise as a flexible, durable, and evolution-resilient antiviral strategy for future outbreaks.

## Conclusion

This study presents a comprehensive evaluation of LNP-encapsulated, chemically modified siRNAs for SARS-CoV-2 therapeutics, incorporating post-infectious therapeutic efficacy, delivery efficiency, and immune compatibility. Among three clinically approved LNP platforms, MC3 formulation exhibited superior delivery efficiency and the least off-target immune activation. Chemical modification with Ome-AS456 effectively suppressed ISG responses while preserving gene silencing and antiviral efficacy of LNP-siRNAs. Importantly, the LNP-siRNAs demonstrated strong therapeutic performance even when administered 24 h post-infection. These findings highlight the potential of LNP-encapsulated, chemically modified siRNAs as a robust and mutation-resilient antiviral strategy. Further studies and optimizations are required to evaluate their therapeutic efficacy in animal models and human respiratory systems to support clinical translation.

## Supplementary Information

Below is the link to the electronic supplementary material.

Supplementary file1 (DOCX 3264 KB)

## Data Availability

All data supporting the findings of this study are available within the paper and its Supplementary Information.

## Abbreviations

SARS-CoV-2: Severe acute respiratory syndrome coronavirus 2
VOC: Variant of concern
MOI: Multiplicity of infection
siRNA: Small interfering RNA
LNP: Lipid nanoparticle
LNP-siRNA: Lipid nanoparticle-encapsulated small interfering RNA
MC3: D-Lin-MC3-DMA
Mem: Membrane
Nuc: Nucleocapsid
Nsp1: ORF1a non-structural protein 1
GFP: Green fluorescent protein
ISG: Interferon-stimulated gene
BALF: Bronchoalveolar lavage fluid
EE (%): Encapsulation efficiency
PI (%): Polydispersity index
FVC: Forced vital capacity
IC: Inspiratory capacity
Rn: Resistance of central airways
Rrs: Resistance of the respiratory system
TLC: Total lung capacity

## References

[1] Synowiec A, Szczepanski A, Barreto-Duran E, Lie LK, Pyrc K. Severe Acute Respiratory Syndrome Coronavirus 2 (SARS-CoV-2): a Systemic Infection. Clin Microbiol Rev. 2021;34(2). 10.1128/CMR.00133-20. PMC7849242 33441314

[2] Jackson LA, Anderson EJ, Rouphael NG, Roberts PC, Makhene M, Coler RN, et al. An mRNA vaccine against SARS-CoV-2 - preliminary report. N Engl J Med. 2020;383(20):1920–31. 10.1056/NEJMoa2022483. PMC7377258 32663912

[3] Counoupas C, Johansen MD, Stella AO, Nguyen DH, Ferguson AL, Aggarwal A, et al. A single dose, BCG-adjuvanted COVID-19 vaccine provides sterilising immunity against SARS-CoV-2 infection. NPJ Vaccines. 2021;6(1):143. 10.1038/s41541-021-00406-4. PMC8633321 34848711

[4] Naaber P, Tserel L, Kangro K, Sepp E, Jurjenson V, Adamson A, et al. Dynamics of antibody response to BNT162b2 vaccine after six months: a longitudinal prospective study. The Lancet Regional Health - Europe. 2021;10:100208. 10.1016/j.lanepe.2021.100208. PMC8418937 34514454

[5] Paxlovid for treatment of COVID-19. Med Lett Drugs Ther. 2022;64(1642):9–10. 35134040

[6] Syed YY. Molnupiravir: first approval. Drugs. 2022. 10.1007/s40265-022-01684-5. PMC8858220 35184266

[7] Vangeel L, Chiu W, De Jonghe S, Maes P, Slechten B, Raymenants J, et al. Remdesivir, Molnupiravir and Nirmatrelvir remain active against SARS-CoV-2 Omicron and other variants of concern. Antiviral Res. 2022;198:105252. 10.1016/j.antiviral.2022.105252. PMC8785409 35085683

[8] Gupta A, Gonzalez-Rojas Y, Juarez E, Crespo Casal M, Moya J, Falci DR, et al. Early treatment for Covid-19 with SARS-CoV-2 neutralizing antibody Sotrovimab. N Engl J Med. 2021;385(21):1941–50. 10.1056/NEJMoa2107934. 34706189

[9] Polkinghorne A, Branley JM. Medications for early treatment of COVID-19 in Australia. Med J Aust. 2022;217(9):S7-13. 10.5694/mja2.51750. PMC9828711 36273391

[10] Yang W, Zhang W, Zhou J, Ma X, Wang C, Zhao M, et al. Comparison of the therapeutic effect of Paxlovid and Azvudine in the treatment of COVID-19: a retrospective study. J Infect Public Health. 2024;17(12):102583. 10.1016/j.jiph.2024.102583. 39500259

[11] Seyedalipour F, Alipour S, Mehdinezhad H, Akrami R, Shirafkan H. Incidence of elevated liver enzyme levels in patients receiving remdesivir and its effective factors. Middle East J Dig Dis. 2024;16(2):109–13. 10.34172/mejdd.2024.377. PMC11316192 39131106

[12] Jinda T, Mizuno S, Tatami S, Kasai M, Ishida T. Risk factors for liver enzyme elevation with remdesivir use in the treatment of paediatric COVID-19. J Paediatr Child Health. 2024;60(7):299–302. 10.1111/jpc.16569. 38818963

[13] Chu WM, Wan EYF, Ting Wong ZC, Tam AR, Kei Wong IC, Yin Chan EW, et al. Comparison of safety and efficacy between Nirmatrelvir-ritonavir and molnupiravir in the treatment of COVID-19 infection in patients with advanced kidney disease: a retrospective observational study. EClinicalMedicine. 2024;72:102620. 10.1016/j.eclinm.2024.102620. PMC11087721 38737003

[14] Petersen JJ, Jorgensen CK, Faltermeier P, Siddiqui F, Feinberg J, Nielsen EE, et al. Drug interventions for prevention of COVID-19 progression to severe disease in outpatients: a systematic review with meta-analyses and trial sequential analyses (The LIVING Project). BMJ Open. 2023;13(6):e064498. 10.1136/bmjopen-2022-064498. PMC10314423 37339844

[15] Torrente-Lopez A, Hermosilla J, Navas N, Cuadros-Rodriguez L, Cabeza J, Salmeron-Garcia A. The relevance of monoclonal antibodies in the treatment of COVID-19. Vaccines. 2021. 10.3390/vaccines9060557. PMC8229508 34073559

[16] Bowden-Reid E, Ledger S, Zhang Y, Di Giallonardo F, Aggarwal A, Stella AO, et al. Novel siRNA therapeutics demonstrate multi-variant efficacy against SARS-CoV-2. Antiviral Res. 2023;217:105677. 10.1016/j.antiviral.2023.105677. 37478918

[17] Iribe H, Miyamoto K, Takahashi T, Kobayashi Y, Leo J, Aida M, et al. Chemical modification of the siRNA seed region suppresses off-target effects by steric hindrance to base-pairing with targets. ACS Omega. 2017;2(5):2055–64. 10.1021/acsomega.7b00291. PMC5808362 29450406

[18] Meng Z, Lu M. RNA interference-induced innate immunity, off-target effect, or immune adjuvant? Front Immunol. 2017;8:331. 10.3389/fimmu.2017.00331. PMC5362589 28386261

[19] Solanki R, Shankar A, Modi U, Patel S. New insights from nanotechnology in SARS-CoV-2 detection, treatment strategy, and prevention. Mater Today Chem. 2023;29:101478. 10.1016/j.mtchem.2023.101478. PMC9981536 36950312

[20] Zhang Y, Johansen MD, Ledger S, Turville S, Thordarson P, Hansbro PM, et al. Respiratory delivery of highly conserved antiviral siRNAs suppress SARS-CoV-2 infection. Int J Mol Sci. 2025. 10.3390/ijms262311675. PMC12692593 41373822

[21] Adams D, Gonzalez-Duarte A, O’Riordan WD, Yang CC, Ueda M, Kristen AV, et al. Patisiran, an RNAi therapeutic, for hereditary transthyretin amyloidosis. N Engl J Med. 2018;379(1):11–21. 10.1056/NEJMoa1716153. 29972753

[22] Walsh EE, Frenck RW Jr., Falsey AR, Kitchin N, Absalon J, Gurtman A, et al. Safety and immunogenicity of two RNA-based Covid-19 vaccine candidates. N Engl J Med. 2020;383(25):2439–50. 10.1056/NEJMoa2027906. PMC7583697 33053279

[23] Hou X, Zaks T, Langer R, Dong Y. Lipid nanoparticles for mRNA delivery. Nat Rev Mater. 2021;6(12):1078–94. 10.1038/s41578-021-00358-0. PMC8353930 34394960

[24] Ferraresso F, Strilchuk AW, Juang LJ, Poole LG, Luyendyk JP, Kastrup CJ. Comparison of DLin-MC3-DMA and ALC-0315 for siRNA delivery to hepatocytes and hepatic stellate cells. Mol Pharm. 2022;19(7):2175–82. 10.1021/acs.molpharmaceut.2c00033. PMC9621687 35642083

[25] Zhu Y, Chidekel A, Shaffer TH. Cultured human airway epithelial cells (calu-3): a model of human respiratory function, structure, and inflammatory responses. Crit Care Res Pract. 2010. 10.1155/2010/394578. PMC2951077 20948883

[26] Harcourt JL, Caidi H, Anderson LJ, Haynes LM. Evaluation of the Calu-3 cell line as a model of in vitro respiratory syncytial virus infection. J Virol Methods. 2011;174(1–2):144–9. 10.1016/j.jviromet.2011.03.027. PMC7112923 21458491

[27] Pires De Souza GA, Le Bideau M, Boschi C, Wurtz N, Colson P, Aherfi S, et al. Choosing a cellular model to study SARS-CoV-2. Front Cell Infect Microbiol. 2022;12:1003608. 10.3389/fcimb.2022.1003608. PMC9634005 36339347

[28] Logan A, Howard CB, Huda P, Kimpton K, Ma Z, Thurecht KJ, et al. Targeted delivery of polo-like kinase 1 siRNA nanoparticles using an EGFR-PEG bispecific antibody inhibits proliferation of high-risk neuroblastoma. J Control Release. 2024;367:806–20. 10.1016/j.jconrel.2024.02.007. 38341177

[29] Escalona-Rayo O, Zeng Y, Knol RA, Kock TJF, Aschmann D, Slutter B, et al. In vitro and in vivo evaluation of clinically-approved ionizable cationic lipids shows divergent results between mRNA transfection and vaccine efficacy. Biomed Pharmacother. 2023;165:115065. 10.1016/j.biopha.2023.115065. 37406506

[30] Zufferey R, Nagy D, Mandel RJ, Naldini L, Trono D. Multiply attenuated lentiviral vector achieves efficient gene delivery in vivo. Nat Biotechnol. 1997;15(9):871–5. 10.1038/nbt0997-871. 9306402

[31] Zufferey R, Dull T, Mandel RJ, Bukovsky A, Quiroz D, Naldini L, et al. Self-inactivating lentivirus vector for safe and efficient in vivo gene delivery. J Virol. 1998;72(12):9873–80. 10.1128/JVI.72.12.9873-9880.1998. PMC110499 9811723

[32] Qin XF, An DS, Chen IS, Baltimore D. Inhibiting HIV-1 infection in human T cells by lentiviral-mediated delivery of small interfering RNA against CCR5. Proc Natl Acad Sci U S A. 2003;100(1):183–8. 10.1073/pnas.232688199. PMC140921 12518064

[33] Chang YC, Yang CF, Chen YF, Yang CC, Chou YL, Chou HW, et al. A siRNA targets and inhibits a broad range of SARS-CoV-2 infections including Delta variant. EMBO Mol Med. 2022;14(4):e15298. 10.15252/emmm.202115298. PMC8988202 35138028

[34] Robbins M, Judge A, Liang L, McClintock K, Yaworski E, MacLachlan I. 2’-O-methyl-modified RNAs act as TLR7 antagonists. Mol Ther. 2007;15(9):1663–9. 10.1038/sj.mt.6300240. 17579574

[35] Petrova Kruglova NS, Meschaninova MI, Venyaminova AG, Zenkova MA, Vlassov VV, Chernolovskaya EL. 2’-O-methyl-modified anti-MDR1 fork-siRNA duplexes exhibiting high nuclease resistance and prolonged silencing activity. Oligonucleotides. 2010;20(6):297–308. 10.1089/oli.2010.0246. 21028964

[36] Ahlenstiel C, Mendez C, Lim ST, Marks K, Turville S, Cooper DA, et al. Novel RNA duplex locks HIV-1 in a latent state via chromatin-mediated transcriptional silencing. Mol Ther Nucleic Acids. 2015;4(10):e261. 10.1038/mtna.2015.31. PMC4881759 26506039

[37] Awad W, Mayall JR, Xu W, Johansen MD, Patton T, Lim XY, et al. Cigarette smoke components modulate the MR1-MAIT axis. J Exp Med. 2025. 10.1084/jem.20240896. PMC11740918 39820322

[38] Danaei M, Dehghankhold M, Ataei S, Hasanzadeh Davarani F, Javanmard R, Dokhani A, et al. Impact of particle size and polydispersity index on the clinical applications of lipidic nanocarrier systems. Pharmaceutics. 2018. 10.3390/pharmaceutics10020057. PMC6027495 29783687

[39] Chow AH, Tong HH, Chattopadhyay P, Shekunov BY. Particle engineering for pulmonary drug delivery. Pharm Res. 2007;24(3):411–37. 10.1007/s11095-006-9174-3. 17245651

[40] Jia M, Han S, Li L, Fu Y, Zhou D. Interferon-stimulated genes: novel targets in renal pathogenesis. Kidney Dis (Basel). 2025;11(1):390–401. 10.1159/000546141. PMC12165645 40519215

[41] Jackson AL, Linsley PS. Recognizing and avoiding siRNA off-target effects for target identification and therapeutic application. Nat Rev Drug Discov. 2010;9(1):57–67. 10.1038/nrd3010. 20043028

[42] Neumeier J, Meister G. SiRNA specificity: RNAi mechanisms and strategies to reduce off-target effects. Front Plant Sci. 2020;11:526455. 10.3389/fpls.2020.526455. PMC7876455 33584737

[43] Tschuch C, Schulz A, Pscherer A, Werft W, Benner A, Hotz-Wagenblatt A, et al. Off-target effects of siRNA specific for GFP. BMC Mol Biol. 2008;9:60. 10.1186/1471-2199-9-60. PMC2443166 18577207

[44] Bowden-Reid E, Moles E, Kelleher A, Ahlenstiel C. Harnessing antiviral RNAi therapeutics for pandemic viruses: SARS-CoV-2 and HIV. Drug Deliv Transl Res. 2025. 10.1007/s13346-025-01788-x. PMC12137459 39833468

[45] Hoy SM. Patisiran: first global approval. Drugs. 2018;78(15):1625–31. 10.1007/s40265-018-0983-6. 30251172

[46] Kelliher MT, Levy JJ, Nerenz RD, Poore B, Johnston AA, Rogers AR, et al. Comparison of symptoms and antibody response following administration of Moderna or Pfizer SARS-CoV-2 vaccines. Arch Pathol Lab Med. 2022;146(6):677–85. 10.5858/arpa.2021-0607-SA. 35188563

[47] Sridhara S, Nair R, Stanek M. Severe aplastic anemia after receiving SARS-CoV-2 Moderna mRNA vaccination. J Hematol. 2022;11(1):34–9. 10.14740/jh954. PMC8929201 35356634

[48] Malayala SV, Mohan G, Vasireddy D, Atluri P. Purpuric rash and thrombocytopenia after the mRNA-1273 (Moderna) COVID-19 vaccine. Cureus. 2021;13(3):e14099. 10.7759/cureus.14099. PMC7996471 33786251

[49] Takase B, Hayashi K, Hisada T, Tsuchiya T, Masaki N, Nagata M. Chest pain with new abnormal electrocardiogram development after injection of COVID-19 vaccine manufactured by Moderna. Intern Med. 2022;61(13):2089–90. 10.2169/internalmedicine.8711-21. PMC9334252 34866106

[50] Dutt D, Lam J, Richards J. Bilateral relentless placoid chorioretinitis following Pfizer-BioNTech COVID-19 vaccination: specific antigenic trigger or nonspecific immune activation? Ocul Immunol Inflamm. 2024;32(7):1498–503. 10.1080/09273948.2023.2239338. 37552853

[51] Shah PP, Gelnick S, Zhu D, Wong A, Verma R. Optic neuritis secondary to the Pfizer-BioNTech-162b2 COVID-19 vaccine managed with plasmapheresis: a case report and review. Oman J Ophthalmol. 2022;15(3):397–402. 10.4103/ojo.ojo_354_21. PMC9905924 36760953

[52] Chachar TS, Yousuf N, Sulaibikh L, Abdulqader F, Alqahtani M. First report of acute myocarditis post-Pfizer-BioNTech COVID-19 vaccination in the Kingdom of Bahrain. Cureus. 2021;13(12):e20313. 10.7759/cureus.20313. PMC8747994 35028213

[53] Sun J, Lu S, Xiao J, Xu N, Li Y, Xu J, et al. Inhibition of SARS-CoV-2 replication by self-assembled siRNA nanoparticles targeting multiple highly conserved viral sequences. Viruses. 2024. 10.3390/v16071072. PMC11281333 39066234

[54] Taibe NS, Mahmoud SH, Kord MA, Badawy MA, Shehata M, Elhefnawi M. In silico and in vitro development of novel small interfering RNAs (siRNAs) to inhibit SARS-CoV-2. Comput Struct Biotechnol J. 2025;27:1460–71. 10.1016/j.csbj.2025.03.034. PMC12008453 40256168

[55] Idris A, Davis A, Supramaniam A, Acharya D, Kelly G, Tayyar Y, et al. A SARS-CoV-2 targeted siRNA-nanoparticle therapy for COVID-19. Mol Ther. 2021;29(7):2219–26. 10.1016/j.ymthe.2021.05.004. PMC8118699 33992805

[56] Zhou Y, Liao Y, Fan L, Wei X, Huang Q, Yang C, et al. Lung-targeted lipid nanoparticle-delivered siUSP33 attenuates SARS-CoV-2 replication and virulence by promoting envelope degradation. Adv Sci. 2024;11(42):e2406211. 10.1002/advs.202406211. PMC11558077 39301916

[57] Idris A, Supramaniam A, Tayyar Y, Kelly G, McMillan NAJ, Morris KV. An intranasally delivered ultra-conserved siRNA prophylactically represses SARS-CoV-2 infection in the lung and nasal cavity. Antiviral Res. 2024;222:105815. 10.1016/j.antiviral.2024.105815. 38246206

[58] Supramaniam A, Tayyar Y, Clarke DTW, Kelly G, Acharya D, Morris KV, et al. Prophylactic intranasal administration of lipid nanoparticle formulated siRNAs reduce SARS-CoV-2 and RSV lung infection. J Microbiol Immunol Infect. 2023;56(3):516–25. 10.1016/j.jmii.2023.02.010. PMC9991324 36934064

[59] Yathindranath V, Safa N, Tomczyk MM, Dolinsky V, Miller DW. Lipid nanoparticle-based inhibitors for SARS-CoV-2 host cell infection. Int J Nanomedicine. 2024;19:3087–108. 10.2147/IJN.S448005. PMC10984206 38562613

[60] Haghi M, Young PM, Traini D, Jaiswal R, Gong J, Bebawy M. Time- and passage-dependent characteristics of a Calu-3 respiratory epithelial cell model. Drug Dev Ind Pharm. 2010;36(10):1207–14. 10.3109/03639041003695113. 20374185

[61] Foster KA, Avery ML, Yazdanian M, Audus KL. Characterization of the Calu-3 cell line as a tool to screen pulmonary drug delivery. Int J Pharm. 2000;208(1–2):1–11. 10.1016/s0378-5173(00)00452-x. 11064206

[62] Kreft ME, Jerman UD, Lasic E, Hevir-Kene N, Rizner TL, Peternel L, et al. The characterization of the human cell line Calu-3 under different culture conditions and its use as an optimized in vitro model to investigate bronchial epithelial function. Eur J Pharm Sci. 2015;69:1–9. 10.1016/j.ejps.2014.12.017. 25555374

[63] Baczenas JJ, Andersen H, Rashid S, Yarmosh D, Puthuveetil N, Parker M, et al. Propagation of SARS-CoV-2 in Calu-3 cells to eliminate mutations in the furin cleavage site of spike. Viruses. 2021. 10.3390/v13122434. PMC8704555 34960703

[64] Li M, Ayyanathan K, Dittmar M, Miller J, Tapescu I, Lee JS, et al. SARS-CoV-2 ORF6 protein does not antagonize interferon signaling in respiratory epithelial Calu-3 cells during infection. mBio. 2023;14(4):e0119423. 10.1128/mbio.01194-23. PMC10470815 37377442

[65] Liu Y, Suzuoki M, Tanaka H, Sakurai Y, Hatakeyama H, Akita H. Lymphatic endothelial cells produce chemokines in response to the lipid nanoparticles used in RNA vaccines. Biol Pharm Bull. 2024;47(3):698–707. 10.1248/bpb.b23-00689. 38538323

[66] Tahtinen S, Tong AJ, Himmels P, Oh J, Paler-Martinez A, Kim L, et al. IL-1 and IL-1ra are key regulators of the inflammatory response to RNA vaccines. Nat Immunol. 2022;23(4):532–42. 10.1038/s41590-022-01160-y. 35332327

[67] Ju Y, Lee WS, Pilkington EH, Kelly HG, Li S, Selva KJ, et al. Anti-PEG antibodies boosted in humans by SARS-CoV-2 lipid nanoparticle mRNA vaccine. ACS Nano. 2022;16(8):11769–80. 10.1021/acsnano.2c04543. 35758934

[68] Estape Senti M, de Jongh CA, Dijkxhoorn K, Verhoef JJF, Szebeni J, Storm G, et al. Anti-PEG antibodies compromise the integrity of PEGylated lipid-based nanoparticles via complement. J Control Release. 2022;341:475–86. 10.1016/j.jconrel.2021.11.042. 34890719

[69] Calmet H, Inthavong K, Eguzkitza B, Lehmkuhl O, Houzeaux G, Vazquez M. Nasal sprayed particle deposition in a human nasal cavity under different inhalation conditions. PLoS One. 2019;14(9):e0221330. 10.1371/journal.pone.0221330. PMC6730903 31490971

[70] Hassett KJ, Benenato KE, Jacquinet E, Lee A, Woods A, Yuzhakov O, et al. Optimization of lipid nanoparticles for intramuscular administration of mRNA vaccines. Mol Ther Nucleic Acids. 2019;15:1–11. 10.1016/j.omtn.2019.01.013. PMC6383180 30785039

[71] Omo-Lamai S, Wang Y, Patel MN, Milosavljevic A, Zuschlag D, Poddar S, et al. Limiting endosomal damage sensing reduces inflammation triggered by lipid nanoparticle endosomal escape. Nat Nanotechnol. 2025;20(9):1285–97. 10.1038/s41565-025-01974-5. PMC12352612 40789922

[72] Parua P, Ghosh S, Jana K, Seth A, Debnath B, Kumar Rout S, et al. Therapeutic potential of neutralizing monoclonal antibodies (nMAbs) against SARS-CoV-2 Omicron Variant. Curr Pharm Des. 2024. 10.2174/0113816128334441241108050528. 39543801

[73] Dong H, Zhou R, Chen J, Wei J, Wei Z, Yang Z, et al. Super broad and protective nanobodies against Sarbecoviruses including SARS-CoV-1 and the divergent SARS-CoV-2 subvariant KP.3.1.1. PLoS Pathog. 2024;20(11):e1012625. 10.1371/journal.ppat.1012625. PMC11554226 39527594

[74] Piepenbrink MS, Khalil AM, Chang A, Mostafa A, Basu M, Sarkar S, et al. Potent neutralization by a RBD antibody with broad specificity for SARS-CoV-2 JN.1 and other variants. Npj Viruses. 2024;2(1):55. 10.1038/s44298-024-00063-z. PMC11564104 39553825

[75] Stein SC, Hansen G, Ssebyatika G, Stroh LJ, Ochulor O, Herold E, et al. A human monoclonal antibody neutralizing SARS-CoV-2 Omicron variants containing the L452R mutation. J Virol. 2024;e0122324. 10.1128/jvi.01223-24. PMC11650997 39494911

[76] Anglero-Rodriguez YI, Lempp FA, Subramanian M, McIninch J, Schlegel MK, Bohan D, et al. High resistance barrier and prophylactic protection in preclinical models of SARS-CoV-2 with two siRNA combination. Nucleic Acids Res. 2025. 10.1093/nar/gkae1195. PMC11724309 39657790

[77] Alarcon-Aris D, Recasens A, Galofre M, Carballo-Carbajal I, Zacchi N, Ruiz-Bronchal E, et al. Selective α-synuclein knockdown in monoamine neurons by intranasal oligonucleotide delivery: potential therapy for Parkinson’s disease. Mol Ther. 2018;26(2):550–67. 10.1016/j.ymthe.2017.11.015. PMC5835151 29273501

[78] Landh E, Moir LM, Traini D, Young PM, Ong HX. Properties of rapamycin solid lipid nanoparticles for lymphatic access through the lungs & part II: the effect of nanoparticle charge. Nanomedicine (Lond). 2020;15(20):1947–63. 10.2217/nnm-2020-0192. 32812483

[79] Strachan JB, Dyett BP, Nasa Z, Valery C, Conn CE. Toxicity and cellular uptake of lipid nanoparticles of different structure and composition. J Colloid Interface Sci. 2020;576:241–51. 10.1016/j.jcis.2020.05.002. 32428785

[80] Xue HY, Liu S, Wong HL. Nanotoxicity: a key obstacle to clinical translation of siRNA-based nanomedicine. Nanomedicine. 2014;9(2):295–312. 10.2217/nnm.13.204. PMC4095781 24552562

[81] Rouet R, Henry JY, Johansen MD, Sobti M, Balachandran H, Langley DB, et al. Broadly neutralizing SARS-CoV-2 antibodies through epitope-based selection from convalescent patients. Nat Commun. 2023;14(1):687. 10.1038/s41467-023-36295-5. PMC9907207 36755042

[82] Khaitov M, Nikonova A, Kofiadi I, Shilovskiy I, Smirnov V, Elisytina O, et al. Treatment of COVID-19 patients with a SARS-CoV-2-specific siRNA-peptide dendrimer formulation. Allergy. 2023;78(6):1639–53. 10.1111/all.15663. 36721963

[83] Tuttolomondo M, Pham STD, Terp MG, Cendan Castillo V, Kalisi N, Vogel S, et al. A novel multitargeted self-assembling peptide-siRNA complex for simultaneous inhibition of SARS-CoV-2-host cell interaction and replication. Mol Ther Nucleic Acids. 2024;35(3):102227. 10.1016/j.omtn.2024.102227. PMC11203390 38939051

[84] Merkel OM. Can pulmonary RNA delivery improve our pandemic preparedness? J Control Release. 2022;345:549–56. 10.1016/j.jconrel.2022.03.039. PMC8958776 35358609

