# Supplementary material for: Comparative analysis of clinically approved lipid nanoparticles for intranasal siRNA delivery against SARS-CoV-2
Source: Drug Deliv Transl Res. 2026 Feb 18;16(10):3848–71. doi: 10.1007/s13346-026-02076-y (PMC13619765; doi:10.1007/s13346-026-02076-y)
Supplement: Supplementary file 1 [file 13346_2026_2076_MOESM1_ESM.docx]

**Supplementary data**

**Figure S1.** **Verification of Calu-3 lentiviral SARS-CoV-2 reporter cell lines**

**(A)** Verification of Calu-3 SARS-CoV-2 lentiviral reporter cell lines. Calu-3 reporter cell lines expressing SARS‐CoV‐2 Nucleocapsid, Membrane, and ORF1a-NSP1 were transfected with 5 nM siRNA 18, siRNA 25, and siRNA 30 (produced by UNSW RNA Institute) using Lipofectamine RNAiMAX (Invitrogen, USA). After 48 hours of transfection, cell fixation with 2% paraformaldehyde and flow cytometry was then performed for GFP expression. Data were presented as mean and standard deviation (SD). All results were normalized with GFP expression of targeted 293T-GFP-reporter cells. Statistical comparisons were performed within each reporter cell line using one-way ANOVA followed by Dunnett’s test to compare each siRNA treatment with its corresponding control. **p<0.005 ****p<0.0001

**Supplementary Figure S2. Size Distribution Profiles of LNP-siRNA Formulations.**

Dynamic light scattering (DLS)–derived size distribution profiles (Intensity % vs Size [nm]) for MC3-, SM-102-, and ALC-0315-based LNP formulations encapsulating siRNA-Scramble (Sc) and siRNAs 18, 25, 27, and 30. Each panel represents triplicate measurements acquired with a Zetasizer Nano (Zetasizer software, Malvern Instruments). All formulations exhibited narrow, single peaks with mean sizes ranging from ~80 to 110 nm.

**Figure S3. Verification of MC3-LNP-siRNAs in Calu-3-reporter cell lines**

GFP suppression and percentage of LNP positive cells of **(A)** MC3-LNP-siRNA 18 (25 nM, 50 nM, and 100 nM) in Calu-3 Mem-GFP reporter cell lines, **(B)** MC3-LNP-siRNA 25(25 nM, 50 nM, and 100 nM) in Calu-3 Nuc-GFP reporter cell lines.and **(C)** MC3-LNP-siRNA 27 (25 nM, 50 nM, and 100 nM) and **(D)** MC3-LNP-siRNA 30 (25 nM, 50 nM, and 100 nM) in Calu-3 ORF1a-NSP1 GFP reporter cell lines. Data were presented as mean and standard deviation, connected by lines for trend visualization only. All experiments were performed in triplicate. Statistical comparisons were performed within each reporter cell line using One-Way ANOVA followed by Dunnett’s test to compare each siRNA treatment with its corresponding LNP-siRNA-Scramble control. * p < 0.05, ** p < 0.01, *** p < 0.001, **** p < 0.0001.

**Figure S4. Verification of SM-102-LNP-siRNAs in Calu-3-reporter cell lines.**

GFP suppression and percentage of LNP positive cells of (A) SM-102-LNP-siRNA 18 (25 nM, 50 nM, and 100 nM) in Calu-3 Mem-GFP reporter cell lines, (B) SM-102-LNP-siRNA 25(25 nM, 50 nM, and 100 nM) in Calu-3 Nuc-GFP reporter cell lines.and (C) SM-102-LNP-siRNA 27 (25 nM, 50 nM, and 100 nM) and (D) SM-102-LNP-siRNA 30 (25 nM, 50 nM, and 100 nM) in Calu-3 ORF1a-NSP1 GFP reporter cell lines. All the results were normalized with LNP-siRNA-Scramble with the corresponding concentrations. Data were presented as mean and standard deviation (SD), connected by lines for trend visualization only. All experiments were performed in triplicate. Statistical comparisons were performed within each reporter cell line using One-Way ANOVA followed by Dunnett’s test to compare each siRNA treatment with its corresponding LNP-siRNA-Scramble control. * p < 0.05, ** p < 0.01, *** p < 0.001, **** p < 0.0001.

**Figure S5. Verification of ALC-0315-LNP-siRNAs in Calu-3-reporter cell lines.**

GFP suppression and percentage of LNP positive cells of (A) ALC-0315 -LNP-siRNA 18 (25 nM, 50 nM, and 100 nM) in Calu-3 Mem-GFP reporter cell lines, (B) ALC-0315 -LNP-siRNA 25(25 nM, 50 nM, and 100 nM) in Calu-3 Nuc-GFP reporter cell lines.and (C) ALC-0315 -LNP-siRNA 27 (25 nM, 50 nM, and 100 nM) and (D) ALC-0315 -LNP-siRNA 30 (25 nM, 50 nM, and 100 nM) in Calu-3 ORF1a-NSP1 GFP reporter cell lines. All the results were normalized with LNP-siRNA-Scramble with the corresponding concentrations. Data were presented as mean and standard deviation (SD), connected by lines for trend visualization only. All experiments were performed in triplicate. Statistical comparisons were performed within each reporter cell line using One-Way ANOVA followed by Dunnett’s test to compare each siRNA treatment with its corresponding LNP-siRNA-Scramble control. * p < 0.05, ** p < 0.01, *** p < 0.001, **** p < 0.0001.

**Figure S6. Assessment of off-target ISG effects of LNP-siRNAs in HeLa-T4+ cells.** HeLa T4+ cells (A-D) were seeded at 4×10^4^/well in 48-well plates and LNPs loaded with siRNA 18 (MC3 18, SM-102 18, and ALC-0315 18), siRNA 25(MC3 25, SM-102 25, and ALC-0315 25), siRNA 27(MC3 27, SM-102 27, and ALC-0315 27) or siRNA 30(MC3 30, SM-102 30, and ALC-0315 30) at 50 nM and were transfected 6 hours after cell seeding. Post-treatment at 72 h, cells were lysed and collected for RNA extraction. RT-qPCR was performed for HeLa T4+ cells; (A) IFIT1, (B) OAS1, (C), Viperin, (D) ISG20, and GAPDH was applied as the housekeeping gene. Relative quantification was determined (2^−ΔΔCt^) and statistical comparisons were performed with Mock, excluding both positive Ctrls, IFN β and Poly (I:C) using One-Way ANOVA followed by Dunnett’s test. Data were presented as mean and standard deviation (SD). * p<0.05, ** = p< 0.01, *** = p< 0.001, **** = p< 0.0001.

*×*

**Figure S7. Assessment of MC3 and ALC-0315-based LNP-siRNA 30mod in Calu-3-Nsp1-GFP reporter cell lines.** (A) GFP expression following treatment with MC3 or ALC-0315-based LNP-OmeAS456-modified-siRNA 30 (25 nM, 50 nM, and 100 nM) on Calu-3 Nsp1-GFP reporter cells from day 3 to day 10. All results were normalized with LNP-siRNA-Scramble using the corresponding concentrations, and (B) Percentage of LNP positive cells following the same treatment and time course in A. Data were presented as mean and standard deviation (SD). All experiments were performed in triplicate. Statistical comparisons were performed against the siRNA-scramble control at matched siRNA concentrations and to compare formulations within each concentration using two-way ANOVA followed by Šídák’s test. * = *p<* 0.05, ** = *p<* 0.01, *** = *p<* 0.001, **** = *p<* 0.0001.

**Figure S8. Protective effect of multiplexed LNP-siRNAs 18, 25, 27, and 30 in Vero E6 cells two hours post-infection.** Cell survival assay of multiplexed (A) MC3 LNP-modified siRNA 30 and (B) ALC-0315-based LNP-modified siRNA 30 against SARS-CoV-2 XBB.1.5 infection in Vero E6 Cells. Vero E6 cells were seeded 2×10^3^/well in 96-well plates, and SARS-CoV-2 XBB.1.5 infection (MOI-0.5) was performed 6 hours after cell seeding. Combination of MC3 -LNP-siRNAs (A- MC3 LNP siRNA 18; B- MC3 LNP siRNA 25; C- MC3 LNP siRNA 27; D- MC3 LNP siRNA 30-OmeAS456-mod) and combinations of ALC-0315 LNP-siRNAs (A- ALC-0315 LNP siRNA 18; B- ALC-0315 LNP siRNA 25; C- ALC-0315 LNP siRNA 27; D- ALC-0315 LNP siRNA 30-OmeAS456-mod) were then added 2 hours post-infection. Each LNP-siRNA in the combination was added in equal amounts, with a total concentration of 25 nM. NucBlue was added to stain live cells 5 days post-infection, and cells were imaged to quantitate the relative cell survial % using the IN Cell Analyzer at day 5 to day 7 post-infection/treatment. All live cell percentages were normalized with the negative control (neither LNP-siRNAs or SARS-CoV-2 were added). All experiments included six replicates. Data were presented as mean and standard deviation (SD). Statistical comparisons were performed with the infected siRNA-Scramble group using One-Way ANOVA followed by Dunnett’s test. * = p < 0.05, ** = p < 0.01, *** = p < 0.001, **** = p < 0.0001.

**Figure S9. Antiviral efficacy of MC3-LNP-siRNAs on Vero E6 and Calu-3 cells 24 hours post-infection of SARS-CoV-2 Omicron XBB.1.5.** (A) (B) Protection of MC3-LNP-siRNAs on Vero E6 cells 24 hours after XBB.1.5 infection. (MOI-0.5) Vero E6 cells were seeded 2×10^3^/well in 96-well plates. SARS-CoV-2 XBB.1.5 infection was then performed 6 hours after Vero E6 seeding. MC3-LNP-siRNAs were then added 24 hours after virus infection. NucBlue was added to stain live cell 5 days after infection, and images were taken for cell survival assay by IN Cell Analyzer at Day 6 (A) and Day 7 (B) post-infection. All the live cell percentages were normalized with Negative control. All experiments were performed with six replicates. (C) (D) Protection of MC3-LNP-siRNAs on Calu-3 cells 24 hours after XBB.1.5 infection. (MOI-0.5) Calu-3 cells were seeded 2×10^4^/well in 96-well plates. SARS-CoV-2 XBB.1.5 infection was then performed 24 hours after Calu-3 seeding. MC3-LNP-siRNAs were then added 24 hours after virus infection. NucBlue was added to stain live cell 5 days after infection, and images were taken for cell survival assay by IN Cell Analyzer at Day 6 (C) and Day 7 (D) post-infection. All live cell percentages were normalized with the negative control. All experiments were performed with six replicates. Data were presented as mean and standard deviation (SD). Statistical comparisons were performed with infected siRNA-Scramble group with the same siRNA concentration using One-Way ANOVA followed by Dunnett’s test. * = *p<* 0.05, ** = *p<* 0.01, *** = *p<* 0.001, **** = *p<* 0.0001.
